# Cumulative viral load as a predictor of CD4+ T-cell response to antiretroviral therapy using Bayesian statistical models

**DOI:** 10.1371/journal.pone.0224723

**Published:** 2019-11-13

**Authors:** Joseph B. Sempa, Theresa M. Rossouw, Emmanuel Lesaffre, Martin Nieuwoudt

**Affiliations:** 1 South African Department of Science and Technology (DST)/ National Research Foundation (NRF) Centre of Excellence in Epidemiological Modelling and Analysis (SACEMA), Stellenbosch University, Stellenbosch, South Africa; 2 Institute for Cellular and Molecular Medicine, Department of Immunology, University of Pretoria, Pretoria, South Africa; 3 Interuniversity Institute for Biostatistics and Statistical Bioinformatics, KU Leuven, Leuven, Belgium; University of Sheffield, UNITED KINGDOM

## Abstract

**Introduction:**

There are Challenges in statistically modelling immune responses to longitudinal HIV viral load exposure as a function of covariates. We define Bayesian Markov Chain Monte Carlo mixed effects models to incorporate priors and examine the effect of different distributional assumptions. We prospectively fit these models to an as-yet-unpublished data from the Tshwane District Hospital HIV treatment clinic in South Africa, to determine if cumulative log viral load, an indicator of long-term viral exposure, is a valid predictor of immune response.

**Methods:**

Models are defined, to express ‘slope’, i.e. mean annual increase in CD4 counts, and ‘asymptote’, i.e. the odds of having a CD4 count ≥500 cells/μL during antiretroviral treatment, as a function of covariates and random-effects. We compare the effect of using informative versus non-informative prior distributions on model parameters. Models with cubic splines or Skew-normal distributions are also compared using the conditional Deviance Information Criterion.

**Results:**

The data of 750 patients are analyzed. Overall, models adjusting for cumulative log viral load provide a significantly better fit than those that do not. An increase in cumulative log viral load is associated with a decrease in CD4 count slope (19.6 cells/μL (95% credible interval: 28.26, 10.93)) and a reduction in the odds of achieving a CD4 counts ≥500 cells/μL (0.42 (95% CI: 0.236, 0.730)) during 5 years of therapy. Using informative priors improves the cumulative log viral load estimate, and a skew-normal distribution for the random-intercept and measurement error results is a better fit compared to using classical Gaussian distributions.

**Discussion:**

We demonstrate in an unpublished South African cohort that cumulative log viral load is a strong and significant predictor of both CD4 count slope and asymptote. We argue that Bayesian methods should be used more frequently for such data, given their flexibility to incorporate prior information and non-Gaussian distributions.

## Introduction

The rollout of effective combination antiretroviral therapy (ART) has markedly improved the survival of sub-Saharan African (SSA) Human Immunodeficiency Virus (HIV)-infected populations [1]. However, resource-limited settings are characterised by the late initiation of ART [2] and limited ongoing immunologic or virologic monitoring [3,4]. The difficulty of recovering CD4 count increases with due to decreasing thymic CD4+ T-cell production [5], and also depends on direct and indirect effects of chronic viral replication [6]. Further, in part due to its recent roll-out in SSA and limited monitoring, the long-term immunological consequences of ART remain incompletely understood [7–9].

There are challenges in the statistical modelling of the relationship of HIV viral load (VL) to CD4 counts. As a result, *mechanistic* models, which make particular assumptions regarding underlying biological processes, have commonly been used for this purpose [10,11]. A previous review of statistical models of immune response to ART in SSA revealed an immense diversity in the methodologies employed [12]. However, particularly two main types of statistical models of immune response following ART initiation were identified. Namely, those quantifying, ‘Slope’ of change in CD4 counts within a specified time after initiation, and ‘Asymptote’, which quantify achieving *or not* a predefined minimal ‘normal’ threshold of CD4 count within a specified time. There was also a sub-type of the above, namely ‘Time-to’ achieving a particular threshold [12]. In this study, we investigate slope and asymptote models.

Cumulative VL (cVL) is a readily definable indicator of long-term *in vivo* exposure to detectable virus [13] and positively associated with an increase in mortality [13]. Few studies have investigated the association of cVL with immune response to ART. Marconi et al [14] used the summated area under a patient’s ‘raw’ VL curve which was then subsequently log-transformed (cVL_1_), as a predictor of immune response in the data of a North American cohort. However, as previously discussed, the methods they employed have statistical weaknesses and the relevance of their findings to resource-constrained SSA settings may be limited [13]. In this study, we use cumulative log VL (cVL_2_), i.e. the summation of the area under the *initially* log-transformed VL curve above the limit of detection.

Previous statistical models have usually assumed normality for CD4 count distributions despite studies demonstrating that this is not justified [12,15–17]. This may have been due to frequentist modelling software requiring this assumption. Bayesian methods, in combination with Markov Chain Monte Carlo (MCMC) computational techniques, allow the use of alternatives to Gaussian distributions, the incorporation of prior information and considerable flexibility with respect to estimation. For a general overview of the Bayesian methodology refer to Lesaffre and Lawson [18] and Gelman et al. [19].

To encourage model reproducibility and standardization, which has been historically lacking, we employ slope and asymptote statistical models based on a consensus of those previously systematically reviewed [12]. Bayesian MCMC mixed-effects methods are used to incorporate priors and examine the effect of different distributional assumptions. We then prospectively apply these models to the data of an as-yet-unpublished SSA HIV treatment cohort and determine if cVL_2_ is a valid predictor of immune response.

## Methods

### Ethics statement

This study was approved by the Research Ethics Committee of the Faculty of Health Sciences at the University of Pretoria (13/2010) and the Tshwane Metsweding Region Research Committee (TMREC 2011/05) to retrospectively capture, and analyse routinely collected data at the Tshwane District Hospital in Gauteng, South Africa. The Research Ethics Committee waived the need for informed consent since the data was de-identified during capture.

### Study setting and patient population

The comprehensive demographic and long-term treatment data of the first, consecutive, 963 patients older than 18 years of age who presented for ART at the Tshwane District Hospital in Gauteng, South Africa during 2004 and 2005 were selected for analysis. All patients started ART after 2004 as part of the South African national HIV treatment plan and were treated according to the National Department of Health HIV guidelines (2004) operative at the time, i.e. eligibility for ART was CD4 <200 cells/μL or WHO stage 4 disease regardless of CD4 count. Treatment was initiated using a standardized triple-drug regimen consisting of two nucleoside reverse transcriptase inhibitors, mostly d4T and 3TC, and one non-nucleoside reverse transcriptase inhibitor, either NVP or EFV. CD4 and HIV-1 VL monitoring was performed at treatment initiation ‘baseline’ and then 6-monthly, according to the national protocol. Demographic, anthropometric, clinical, ART and 5-year longitudinal treatment response data were collected. We excluded all second-line ART visits for all patients who were switched to second-line therapy.

### Data collection and inclusion criteria

Data was collated in Microsoft Excel spreadsheets. Of an initial 963 patients, 213 patients were excluded as they only had a baseline visit, preventing the calculation of cVL_2_. Of the remaining 750 patients, 59 patients had missing data for either sex, baseline CD4 count, baseline log VL or longitudinal CD4 counts. The Bayesian models included imputation for missing data in both the CD4 outcomes and the covariates, during each model iteration [18]. For the ‘slope’ model the data of 750 patients was analysed. In the ‘asymptote’ model, a further 5 patients were excluded as they had CD4 counts ≥500 cells/μL at baseline, leaving 745 patients, see Figure A in S2 Appendix.

### Statistical methods

#### Model definitions

In this study, two types of longitudinal statistical models were investigated. These were defined based on a consensus from a prior systematic review [12]. The utility of including cVL_2_ as a covariate was examined in both cases:

1. The ‘Slope’ model, in which the outcome was the mean annual increase in absolute CD4 count measured at 6-monthly intervals following ART initiation. In this case, the post-ART immune *trajectory* was the metric of interest; and,
2. The ‘Asymptote’ model, in which the outcome was the odds of achieving a CD4 count ≥500 cells/μL at any particular time during the 5 years of ART. If at any visit following ART initiation there was a CD4 count ≥500 cells/μL it was coded as ‘1’, or ‘0’, if below, i.e. a binary outcome. In this case, achieving or not a specified ‘normal’ CD4 count *threshold* was the metric of interest. Note, a 500 cells/μL threshold was chosen as it is the described lower end of the healthy reference range of CD4 counts for the SSA population [20]. Patients were also excluded when their baseline CD4 count was ≥500 cells/μL, as prior studies have shown that when such people initiate ART they have an almost equal life expectancy to the general population [21,22].

The lower detection limit for the VL assay used in this cohort was 50 copies/mL. However, a value of 400 copies/mL was used in the calculation of cVL_2_ [13] as doing so enabled the exclusion of smaller ‘viral blips’ (50–400 copies/mL) that are unrelated to subsequent viral rebound and failure [23,24]. Transitory values of below 1000 copies/mL have been shown to have no quantitative difference in estimates of opportunistic infections and mortality compared to a threshold of 400 copies/mL during treatment [13].

Thus, cVL_2_, measured as copy-year/mL, for the *i*-th patient at their *j*-th visit is given by,

$$cVL_{2ij}=\sum_{k=2}^{k=j}\left(t_{i,k}-t_{i,k-1}\right)\times \frac{\left(log_{10}\left(\frac{V_{i,k}}{400}\right)+log_{10}\left(\frac{V_{i,k-1}}{400}\right)\right)}{2}$$ (1)

where *t*_*i*,*k*_ and *V*_*i*,*k*_ represent the time (in years) and log VL measurement of the *k*-th visit, respectively. cVL_2_ was set to 0 at baseline. All visits where VL was missing (49% of 8250 visits see Figure B in S2 Appendix) were excluded during estimation of cVL_2_. The cVL_2_ for patients with missing VL intervals was estimated with a longer inter-visit duration i.e. (*t*_*i*,*k*_−*t*_*i*,*k*−1_) as seen in *Eq 1*.

#### Variables

These included: sex (‘0’ for males and ‘1’ for females), baseline age (years), baseline CD4 count, baseline log VL (log_10_ copies/mL), time on treatment (years) as per prior model consensus [12], and cVL_2_ (log_10_ copy-year/mL) as described above (*Eq 1*). cVL_2_ coefficient was multiplied with a binary variable (0 –if the patient was lost to follow-up and 1 –if the patient was still in care) (see model implementation in S1 Appendix).

### Model structure

Also as per prior consensus [12], we used linear mixed-effects models, with random intercept and slope on time on treatment, as these models have most often been used to study CD4 count response while on ART [25–27]. Absolute CD4 counts are known to vary within and between patients [8,28] and CD4 measurements further apart in time were found to be less correlated than those closely adjacent. Nash et al. 2008 [22] have demonstrated, in patients initiating ART with <200 cells/μL, that the largest gains in CD4 count were seen in the first 6 months of treatment after which it slowed down considerably. The majority (91.7%) of our cohort had baseline CD4 counts <200 cells/μL.

We define 9 models, each with two scenarios, namely, unadjusted (i.e. without cVL_2_) and adjusted (i.e. with cVL_2_). These are labelled as follows:

- **Model 1:** *slope model* second-degree polynomial model where errors and random effects are normally distributed;
- **Model 2:** *slope model* where errors and random effects are normally distributed and has cubic splines with 3 inner knots;
- **Model 3:** *slope model* where errors and random effects are normally distributed and has cubic splines with 5 inner knots;
- **Model 4:** *slope model* where random-effects are SN distributed;
- **Model 5:** *slope model* where measurement error and random-effects are SN distributed.
- **Model 6:** *Asymptote model* second-degree polynomial model where random effects are normally distributed;
- **Model 7:** *Asymptote model* where random effects are skew-normally distributed;
- **Model 8:** *Asymptote model* where random-effects are normally distributed with cubic splines and 3 inner knots; and
- **Model 9:** *Asymptote model* where random-effects are normally distributed with cubic splines and 5 inner knots.

Slope models (*Eq 2*) were initially defined as second-degree polynomials, i.e. a quadratic term of time on treatment with a random intercept and slope. This was referred to as model 1 and defined as follows:

$$\{\begin{array}{c} y_{i}=x_{i}\;\beta +z_{i}\;b_{i}+\varepsilon_{i},\\ b_{i}∼N\left(0,\Sigma_{b}\right),\;\varepsilon_{i}∼N\left(0,\sigma_{\varepsilon }^{2}I_{m_{i}}\right) \end{array}$$ (2)

for *i* = (1,…,*n*) patients. The CD4 count response is given by an *m*_*i*_-dimensional vector, $y_{i}={\left(y_{i1},y_{i2},\ldots ,y_{im_{i}}\right)}^{T}$, ***β*** = (*β*_0_,*β*_1_,…,*β*_*p*_)^*T*^ is a (*p*+1)−dimensional vector of unknown regression coefficients, $x_{i}={\left(x_{i1},x_{i2},\ldots ,x_{{im}_{i}}\right)}^{T}$ is a (*m*_*i*_ *x*(*p*+1)) matrix of *p* fixed effects, $z_{i}={\left(z_{i1},z_{i2},\ldots ,z_{{im}_{i}}\right)}^{T}$ is a (*m*_*i*_ *x q*) design matrix for the *i*^th^, *q* dimensional vector of random effects. The measurement error $\varepsilon_{i}={\left(\varepsilon_{i1},\varepsilon_{i2},\ldots ,\varepsilon_{im_{i}}\right)}^{T}$ is a *m*_*i*_−dimensional normally distributed vector with mean zero and covariance, $\sigma_{\varepsilon }^{2}I_{m_{i}}$, and assumed to be independent of the random effects. ***b***_*i*_ is assumed to have a bivariate Gaussian distribution with mean zero and (*q x q*) covariance matrix Σ_*b*_.

The model for the response of the *i*-th subject at the *j*-th time point is then given by,

$$y_{ij}∼N(x_{ij}^{T}\;\beta +z_{ij}^{T}\;b_{i},\sigma_{\varepsilon }^{2}).$$

We also examined replacing 2^nd^ degree polynomials with cubic B-splines with 3 inner knots. This procedure slices the data around important time points as previously described by Corbeau et al 2011 [29], at (1.250, 2.5, 3.751), or 5 inner knots, at (0.833, 1.666, 2.5, 3.334, 4.167), that latter of which is simply a more extreme case of the 3 inner knots. For more details, refer to the ‘Spline models’ *model 2 and model 3* in S1 Appendix.

### Distributional considerations

Models such as the above assume that measurement errors and random effects are normally distributed, meaning that responses must also be normally distributed given the covariates. Most prior studies have assumed that CD4 count response is either Gaussian normally distributed or have applied log or square-root variable transformations to achieve *normalization* [30,31]. In isolated cases, the Poisson distribution has also been applied [32]. However, the latter assumes equal mean and variance, which is invariably unjustified. In this case, these parameters differed dramatically, 366 cells/μL versus 56481.3 cells/μL, respectively. Further, at any particular time during treatment, the distribution of CD4 count values are usually skewed [33]. Thus, inferences based on using such methods might result in bias.

For this reason, in addition to the Gaussian normal, we also investigated Skew-normal (***SN***) distributions. The ***SN*** distribution is an extension of the former, consisting of a Gaussian component and a skewness component. Initially, only the random-effects were assumed to have a ***SN*** distribution (*Eq 3*), i.e. ***b***_*i*_~***SN***_*q*_(0,*Σ*_*b*_,Δ_*b*_), where each *i*^th^ individual has a *2*-dimensional random-effects covariance matrix, *Σ*_*b*_. The deviation from normality is given by the skewness part, here chosen the same for all random effects, namely Δ_*b*_ = *diag*(*δ*,…,*δ*). Note that for *δ* = 0 we obtain the Gaussian distribution. For more details, refer to [33,34].

$$\{\begin{array}{c} y_{i}=x_{i}\;\beta +z_{i}\;b_{i}+\varepsilon_{i},\\ b_{i}∼SN_{q}\left(0,\Sigma_{b},\Delta_{b}\right),\;\varepsilon_{i}∼N\left(0,\sigma_{\varepsilon }^{2}I_{m_{i}}\right) \end{array}$$ (3)

Thereafter, ***SN***s were assumed for both random effects and measurement error (*Eq 4*). Namely, $\varepsilon_{i}∼{SN}_{m_{i}}(0,\sigma_{\varepsilon }^{2}I_{m_{i}},\Delta_{\varepsilon_{i}}),$ and random-effects, ***b***_*i*_~***SN***_*q*_(0,*Σ*_*b*_,Δ_*b*_) where $\Delta_{\varepsilon_{i}}$ and Δ_*b*_ are the respective skewness terms. An unknown (*m*_*i*_*x m*_*i*_) skewness diagonal matrix, $\Delta_{\varepsilon_{i}}=diag(\delta_{i1},\delta_{i2},\ldots ,\delta_{im_{i}})$ with skewness parameter vector $\delta_{i}={\left(\delta_{i1},\delta_{i2},\ldots ,\delta_{im_{i}}\right)}^{T}$ was used. Thus:

$$y_{ij}|b_{i},\beta ,\sigma_{\varepsilon }^{2},\Delta_{\varepsilon_{i}}∼SN_{m_{i}}\left(x_{ij}^{T}\beta +z_{ij}^{T}b_{i},\sigma_{\varepsilon }^{2}I_{m_{i}},\Delta_{\varepsilon_{i}}\right)$$ (4)

For more details, refer to the ‘***SN*** models’ *model 4 and model 5* in S1 Appendix

The asymptote models (*Eq 5*) were also initially defined as second-degree polynomials, and the same variables used in the slope model were adjusted for. This was referred to as model 6 and defined as follows:

$$\{\begin{array}{c} logit\left(\pi_{i}\right)=x_{i}\;\beta +z_{i}\;b_{i},\\ b_{i}∼N_{q}\left(0,\Sigma_{b}\right) \end{array}$$ (5)

For *π*_*ij*_ = *prob*(*y*_*ij*_ = 1) if CD4 count ≥500 cells/μL and ‘0’ otherwise for patient ‘*i*’ at visit ‘*j’*. $\pi_{i}={\left(\pi_{i1},\pi_{i2},\ldots ,\pi_{im_{i}}\right)}^{T}$ is an *m*_*i*_-dimensional vector, with a Bernoulli distribution. The model for the *i*^th^ subject at the *j*-th time point is then written as,

$$logit(\pi_{ij})=x_{ij}^{T}\;\beta +z_{ij}^{T}b_{i}.$$

A ***SN*** distribution for the random-effects (*Eq 6*) was also investigated, see ‘***SN*** model’ *model 7* S1 Appendix.

$$\{\begin{array}{c} logit\left(\pi_{i}\right)=x_{i}\;\beta +z_{i}\;b_{i},\\ b_{i}∼SN_{q}\left(0,\Sigma_{b},\Delta_{b}\right) \end{array}$$ (6)

where elements of ***b***_*i*_ are defined in the same way as those in *Eq 3*. Further, we also investigated the use of cubic B-splines with 3 and 5 inner knots, as described for *Eq 2*, refer to ‘Spline models’ *model 8 and model 9* in S1 Appendix.

### Missing data imputation

In the Bayesian framework, missing data imputation uses the data augmentation algorithm [35]. Given dataset ***D*,** with missing data ***MD*,** and parameters ***K***, the algorithm determines, with each iteration, plausible values by sampling ***MD*** from a conditional distribution *P(****MD | D*, *K****)*, and then imputes these values. It then samples ***K*** from a conditional distribution of the posterior *P(****K | D*, *MD****)* [18]. For missing data on baseline CD4 count and baseline VL we estimated *P(****MD | D*, *K****)* using multiple linear regression. For imputation of sex we used multiple logistic regression.

The linear regression imputation models were defined as:

$$\{\begin{array}{c} y_{i}=x_{i}\;\phi +\varepsilon_{i},\\ \varepsilon_{i}∼N\left(0,\omega^{2}\right), \end{array}\;\left(i=1,\ldots ,n\right)$$ (7)

In *Eq 7* the response, CD4 count is an *n*-dimensional vector ***y***_*i*_ = (*y*_1_,*y*_2_,…,*y*_*n*_)^*T*^, assumed to have a linear relationship with ***x***_*i*_ = (1,***x***_**1**_,***x***_**2**_)^*T*^ an (*n x* 3) matrix of 2 fixed effects of sex and baseline age. ***ϕ***^*T*^ = (*ϕ*_0_,*ϕ*_1_,*ϕ*_2_) is a 3-dimensional vector of regression coefficients and ***ε*** an *n*×1 vector of random errors, for additional details refer to S1 Appendix.

$$logit\left(\pi_{i}\right)=x_{i}\;\phi ,\;i=\left(1,\ldots ,n\right)$$ (8)

In Eq 8, for *π*_*i*_ = *prob*(*y*_*i*_ = 1) if sex is female and ‘0’ otherwise for patient ‘*i*’. ***π***_*i*_ = (*π*_1_,*π*_2_,…,*π*_*n*_)^*T*^ is an *m*_*i*_-dimensional vector, with a Bernoulli distribution. ***ϕ***^*T*^ = (*ϕ*_0_,*ϕ*_1_,*ϕ*_2_,*ϕ*_3_) a 4-dimensional vector of regression coefficients and ***x***_*i*_ = (1,***x***_**1**_,***x***_**2**_,***x***_**3**_)^*T*^ an (*n x* 4) matrix of 3 fixed effects of baseline age, baseline CD4 count, and baseline log VL. Note, baseline CD4 count and baseline log VL, which had ***MD***, was used to impute missing sex. For this reason we used *Eq 7* to impute the ***MD*** for the first two variables, and then imputed a value for the missing sex.

#### Priors

**Non-informative priors:** For slope of CD4 count models in *Eqs 2, 3* and *4*, the unknown parameters are: $\left\{\beta ,\sigma_{\varepsilon },\Delta_{\varepsilon_{i}},\rho ,\phi ,\theta ,\tau_{\varepsilon },\sigma_{0},\sigma_{1},\Delta_{b},\vartheta_{b},\gamma_{b},\alpha \right\}$, where for those not defined in the preceding sections, *ρ* is the correlation for the random-effects, ***τ***_***ε***_ is a vector for the scale parameter for the imputed models, *σ*_0_ and *σ*_1_ are the respective standard deviations of the random intercept and slope, ***ϑ***_*b*_ is a vector of parameters that make up the skewness parameter for the random effects in the ***SN*** models, ***γ***_***b***_ is a vector of shape parameters for the random-effects in the covariance matrix of the ***SN*** models, and ***α*** is a vector of coefficients on the random-effects part in both types of ***SN*** models (i.e. *Eqs* 3 and 4). For more details regarding these priors, please refer to the section on priors for each of the models in S1 Appendix.

For asymptote models in *Eqs 5 and 6*, the unknown parameters are: {***β***,*ρ*,***ϕ***,***θ***,***τ***_***ε***_,*σ*_0_,*σ*_1_,***α***,Δ_***b***_,***ϑ***_*b*_, ***γ***_***b***_}, where *ρ* is the correlation for the random-effects, ***τ***_***ε***_ is a vector for the scale parameter for the imputed models, *σ*_0_ and *σ*_1_ are the respective standard deviations of the random intercept and slope, ***ϑ***_*b*_ is a vector of parameters that make up the skewness parameter for the random effects in the ***SN*** models, ***γ***_***b***_ is a vector of shape parameters for the random-effects in the covariance matrix in the ***SN*** models and ***α*** is a vector of coefficients on the random-effects part in of ***SN*** random-effects model. For details about these priors please refer to the section on priors for each of the models in S1 Appendix.

Vague Gaussian, uniform, inverse-gamma, and inverse-Wishart prior distributions were used for the regression coefficients, the error variance and the covariance matrix of the random effects, respectively.

**Informative priors:** For the regression coefficients of sex, baseline log VL, and polynomial terms, the mean and variances were estimated from prior studies [25,36]. Meaning, these were only possible for polynomial models in which the random effects had Gaussian distributions. ***SN*** distributions were not applied in the reviewed prior studies [12]. Simple linear mixed-effect models were generated for each of the covariates—for example, CD4 count as the outcome adjusted for sex as the only covariate, resulting in a posterior estimate for sex (i.e. 36.17). The change in the posterior estimates from the full model, ***model 1*** (i.e. 24.1) to that in the simple linear mixed-effect model (i.e. 36.17) was obtained using this method. This change was then added (i.e. -12.04) to the historical study estimate (i.e. 35.2) [36] to get to 23.16, which was then used as an informative prior mean for sex, see Table 1. The precision was estimated from the variance, *s*^2^, using the formula for the upper 95% confidence intervals (uCI):

uCI=x¯+1.96*(s2n) (9)

where $\bar{x}$ is the mean estimate and ‘*n*’ the particular historical study size. Then, solving for *s*^2^ in *Eq 9*:

$$s^{2}=n\times {\left(\frac{uCI-\bar{x}}{1.96}\right)}^{2}$$ (10)

**Table 1:** Estimating informative priors used for slope of CD4 count model.

Variable	Model Coefficients ^a^					Informative priors		
	Historical study		Current study (Est.)	*model 1* (Est.)	Change^b^	Coef.^c^	Variance (Var.)	Precision (1/Var.)
	Est.	Upper CI						
**Female-sex** [36] ***n = 459***	35.2	66.5	36.17	24.1	-13.17	23.16	117055	8.54E-06
**Baseline log10 viral load (copies/mL)** [36] ***n = 459***	13.9	22.2	-10.09	7.5	-11.89	11.31	8231.08	1.22E-4
**Time on treatment (years)** [25] ***n = 12946***	65	69	56.18	52.3	-0.68	61.12	53919.2	1.86E-05
**Time on treatment (years)-squared** [25] ***n = 12946***	-6	-5	-22.97	-23.0	-0.27	-6.03	3369.95	2.97E-04

^**a**^ list of coefficients from historical simple linear mixed-effects models as published in the respective articles (historical study) and from the same reanalysed models using our data (current study Est.)

^**b**^ obtained by subtracting current study coefficients from full model (***model 1***)

^**c**^ obtained by adding both Change and historical study Est. columns.

For example, in [36] the simple regression coefficient for female-sex and the *uCI* were 35.2 and 66.5 respectively, with a sample size *n* = 459. Substituting this into *Eq 10* yields *s*^2^ = 117054.8, and a precision of 8.543007E-06 (Table 1). In the asymptote models, historical studies used different CD4 count thresholds [12], meaning that informative priors were not possible.

### Data analysis

R version 3.2.2 (R Foundation for Statistical Computing, Vienna, Austria, https://cran.r-project.org/) and OpenBUGS (http://www.openbugs.net) were used. Except for time on treatment, all continuous covariates were standardized to a mean of zero and standard deviation equal to one. Three MCMC chains were employed in both models. For the second-degree polynomial slope models, a burn-in of 20K followed by a further 45K iterations was used. For other variations of the slope and asymptote models, a burn-in of 80K followed by 100K iterations was used. The ***SN*** random-effects slope model had 250K burn-in followed by 250K iterations while that for asymptote model employed 500K burn-in followed by 500K iterations. We ensured that the models ran until the Monte Carlo error was <5% of the posterior standard deviation. For confirmation of convergence, the Brooks-Gelman-Rubin (BGR) diagnostic was used.

Models were selected based on the conditional Deviance Information Criterion (cDIC) [37]. Unless models differ by at least 5, no clear model choice can be expected. Moreover, Quintero and Lesaffre (2018) showed that the marginal DIC (mDIC) should be chosen, based on the marginal specification of the linear mixed-effects model [38]. Unfortunately, there is no software yet available to compute the mDIC in the general case considered here. Hence, we considered only a clear model choice if the cDIC’s differ by approximately 10 [38]. Parameter means and 95% equal-tail credible intervals (95%CI) are reported below.

## Results

### Descriptive results

213 patients were excluded from analysis as they only had a baseline visit. This group had higher median baseline log VLs, i.e. 5.3 log10 copies/mL (IQR: 4.76, 5.54) vs 5.1 log10 copies/mL (IQR: 4.64, 5.48), (p = 0.003), and were older, i.e. 39 years (IQR: 34, 47) vs 36 years (IQR: 31, 42), (p<0.001).

In the data of the 750 patients analysed, 69.6% (n = 522) were female, with a median baseline age of 36 years (IQR: 31, 42). The median baseline CD4 count was 89 cells/μL (IQR: 43, 143) and median baseline log VL, 5.1 log_10_ copies/mL (IQR: 4.64, 5.48). The overall median increase in cVL_2_ after 5 years on ART was 0.3 log_10_ copy-year/mL (IQR: 0.30, 0.43). Patients initiated on ART with baseline CD4 count >200 cells/μL (n = 43) experienced a 278 cells/μL median increase, starting at 261 cells/μL (IQR: 219, 298) and ending at 529 cells/μL (IQR: 420, 633) during 5-years of follow-up. Those with CD4 count 101–200 cells/μL (n = 291) at baseline experienced a 241 cells/μL median increase during 5-years of follow-up on ART from 143 cells/μL (IQR: 120, 173) to 386 cells/μL (IQR: 253, 525). Patients with ≤100 cells/μL (n = 416) at baseline experienced a 270 cells/μL median increase in CD4 count during 5-years of follow-up on ART from 47 cells/μL (IQR: 20, 72) to 317 cells/μL (IQR: 188, 496).

Patients with baseline log VL >5 log_10_ copies/mL (n = 411) experienced a CD4 count median increase of 268 cells/μL during follow-up, from 78 cells/μL (IQR: 41, 131) to 367 cells/μL (IQR: 232, 515), while those with ≤5 log_10_ copies/mL (n = 339) had a 237 cells/μL median increase in CD4 count from 103 cells/μL (IQR: 47, 161) to 365 cells/μL (IQR: 226, 512). The overall median increase in cVL_2_ from ART start by baseline CD4 count category was 0.6 log_10_ copy-year/mL (IQR: 0.44, 0.82), 0.7 log_10_ copy-year/mL (IQR: 0.50, 0.90), and 0.7 log_10_ copy-year/mL (IQR: 0.55, 1.28) for patients with baseline CD4 count >200, 101–200, and ≤100 cells/μL, respectively. Only 33.8% of the patients in the data used in the asymptote model ever reached a CD4 count ≥500 cells/μL.

### Slope models

**Fig 1:**
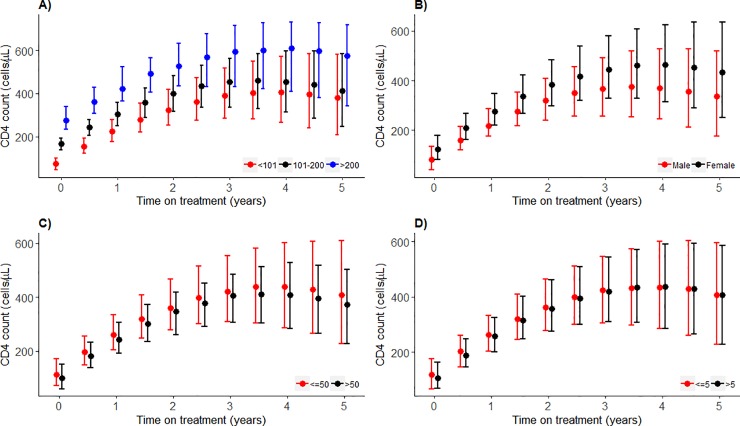
Predicted posterior median CD4 counts trajectory by covariate strata for the slope of CD4 count model, with 95% prediction intervals. A. baseline CD4 count *(cells/μL)*, B. sex, C. baseline age *(years)*, D. baseline log_10_ VL *(copies/mL)*. Median predicted CD4 counts from *model 1*, is plotted. The red, black or blue dots represent predicted median CD4 count at different time points, and the bars are the whiskers.

After adjusting for sex, baseline age, baseline CD4 count, baseline log VL and time on treatment an increase in cVL_2_ is associated with a -19.6 cells/μL (95%CI: -28.25, -10.93) mean annual decrease in absolute CD4 counts over 5-years of follow up, Table 2. All other estimates of the regression coefficients were associated with CD4 count response as their 95%CIs do not include zero. The adjusted, i.e. *with* cVL_2_, model has a substantially lower cDIC compared to the crude, i.e. *without* cVL_2_ (47480 vs 47510, respectively), Table H in S2 Appendix. The median predicted CD4 count response, generated from the cVL_2_ adjusted *model 1*, among patients with baseline CD4 count ≤100 cells/μL is lower compared to those with >200 cells/μL, especially within the first 2 years of ART. The trend is similar but still lower compared to those in the 101–200 cells/μL category. This is more pronounced towards the 5^th^ year of ART, Fig 1A. Females demonstrate a marginally higher median predicted increase in CD4 counts than males, Fig 1B. There is no difference in median predicted CD4 count increase between stratified groups of baseline age (≤50 vs >50 years) or baseline log10 VL (≤5 vs >5 copies/mL) during follow-up, Fig 1C and 1D.

**Table 2:** Posterior means and 95% credible intervals of the regression coefficients for the slope of CD4 count models.

Parameter	*without cVL*_*2*_ *model 1*		*with cVL*_*2*_ *model 1*	
	Estimate	95% CI	Estimate	95% CI
**Female-sex**	24.1	(12.97, 35.29)	23.8	(12.53, 34.96)
**Baseline age**	-6.9	(-12.06, -1.65)	-6.3	(-11.57, -1.08)
**Baseline CD4 count**	82.4	(76.94, 83.10)	82.9	(77.4, 88.38)
**Baseline log viral load**	7.5	(-2.16, 12.92)	7.5	(2.03, 12.92)
**Time on treatment**	52.3	(45.52, 58.98)	55.7	(48.83, 62.65)
**Time on treatment-squared**	-23.0	(-24.99, -20.93)	-22.7	(-24.86, -20.78)
**cumulative log viral load**	–	–	**-19.6**	**(-28.25, -10.93)**

cVL_2_—cumulative HIV log viral load.

### Asymptote models

**Fig 2:**
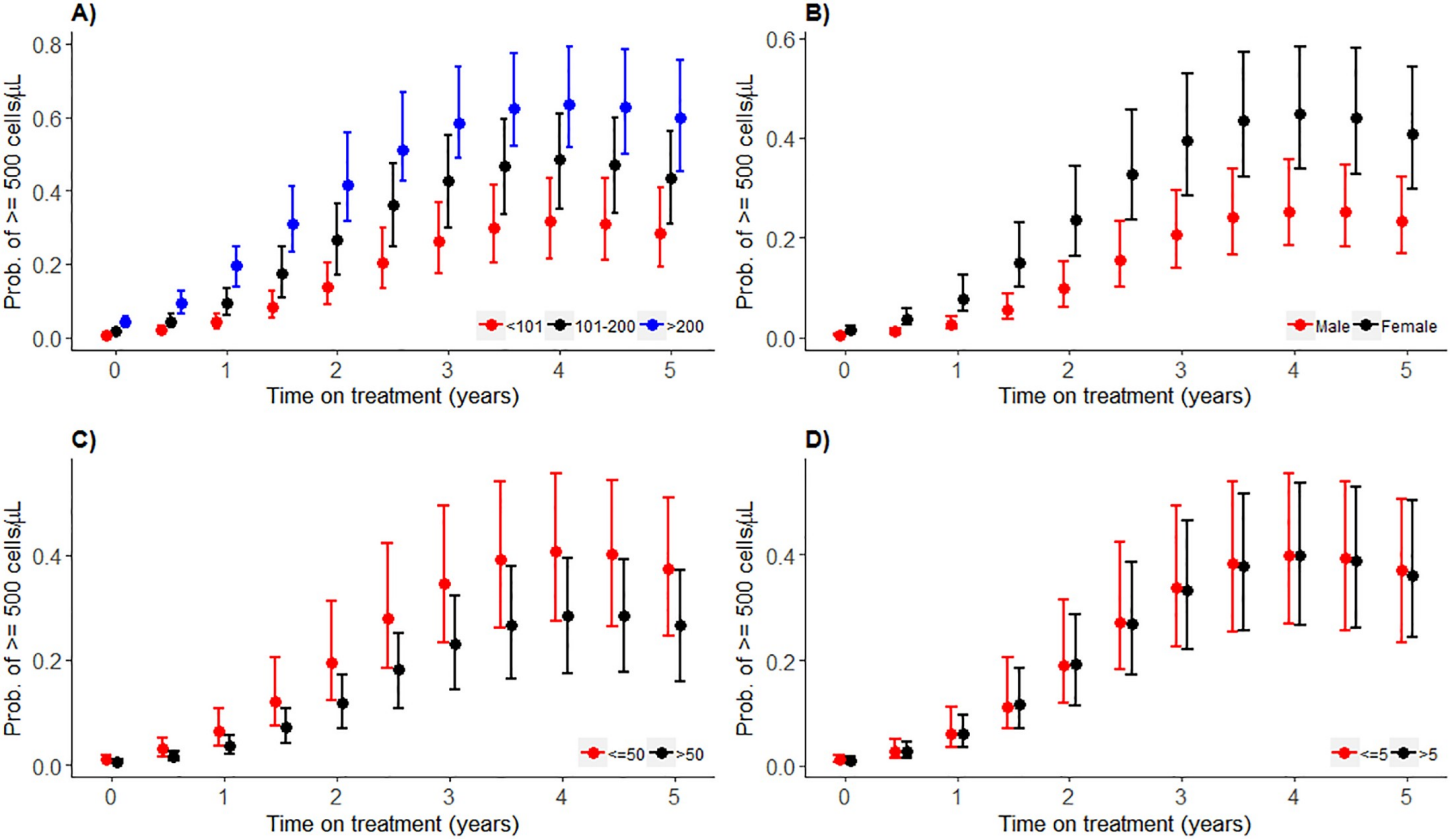
Predicted posterior median probability of having ≥500 cells/μL CD4 counts by covariate strata, with 95% prediction intervals. A) baseline CD4 count (*cells/μL*), B) sex, C) Baseline age (*years*), D) baseline log_10_ viral load (*copies/mL*). Median predicted probability of having CD4 count ≥500 cells/μL from *model 6*. The red, black or blue dots represent predicted probability of having CD4 count ≥500 cells/μL at different time points, and the bars are the whiskers.

After adjusting for sex, baseline age, baseline CD4 count, time on treatment and baseline log VL, an increase in cVL_2_ reduces the odds for having a CD4 count ≥500 cells/μL at any time during 5 years of follow-up, i.e. 0.42 (95%CI: 0.236, 0.730), Table 3. For none of the variables, other than baseline log VL, the 95%CI for the posterior mean odds ratio includes one. The cVL_2_ adjusted compared to the unadjusted *model 6* has a substantially lower cDIC (1695 versus 1716, respectively), Table H in S2 Appendix. Adjusting for cVL_2_ clearly provides a better fit to the data. Except for patients whose baseline CD4 count was >200 cells/μL and 101–200 cells/ μL, the median predicted probability of having a CD4 count ≥500 cells/μL is always less than 0.5 throughout the 5 years of ART, Fig 2A. Females demonstrate a higher median predicted probability of having a CD4 count ≥500 cells/μL compared to males, Fig 2B, especially during the first 2.5 years of treatment. Patients with a baseline age ≤50 years old have similar median predicted probabilities of a CD4 count ≥500 cells/μL compared to those >50 years, Fig 2C. It seems more likely for ≤50-year-old patients to reach at least 0.5 probability of achieving CD4 count ≥500 cells/μL (at 3 years of ART) compared to those >50 years who never reach this during 5 years of follow-up. Patients with higher baseline log_10_ VL, ≤5 copies/mL, have a similar median predicted probability compared to those with baseline log VL >5 copies/mL of having CD4 counts ≥500 cells/μL for 5 years, Fig 2D.

**Table 3:** Posterior mean odds ratios and 95% credible intervals of the regression coefficients for the binary longitudinal models with response: CD4 counts ≥500 cells/μL.

Parameter	*without cVL*_*2*_ *model 6*		*with cVL*_*2*_ *model 6*	
	Estimate	95% CI	Estimate	95% CI
**Female-sex**	5.85	(2.886, 11.977)	6.35	(3.019, 14.382)
**Baseline age, years**	0.55	(0.398, 0.749)	0.54	(0.389, 0.740)
**Baseline CD4 count**	2.82	(2.103, 3.811)	3.00	(2.175, 4.145)
**Baseline log viral load**	1.24	(0.895, 1.705)	1.26	(0.905, 1.799)
**Time on treatment**	3.35	(2.413, 4.457)	4.08	(3.019, 5.579)
**Time on treatment—squared**	0.68	(0.581, 0.814)	0.65	(0.550 0.745)
**cumulative log viral load**	–	–	**0.42**	**(0.236, 0.730)**

cVL_2_—cumulative HIV log viral load.

### Refinements of initial models

In the slope model, changing model structure from a second-degree polynomial to cubic splines, *model 2* and *model 3*, provides significantly higher cDIC’s (Table H in S2 Appendix), indicating that the polynomial model, i.e. *model 1* is a better fit. The random effects are not convincingly normally distributed, Figure C in S2 Appendix, prompting further investigation into ***SN*** distributions. The ***SN*** distribution of the random-effects yields a significantly lower cDIC for both the unadjusted and adjusted, *model 4*, compared to Gaussian models. This is more pronounced in the unadjusted versus adjusted in *model 4* (i.e. 47270 vs 47480, respectively). However, for *model 5* both unadjusted and adjusted models (i.e. with ***SNs*** for both random-effects and measurement errors) have the smallest cDIC’s. Except for the spline models, cVL_2_ remains a significant covariate in determining the slope of CD4 count, Table H in S2 Appendix and Table 4.

**Table 4:** The effect of changing from a Gaussian to a skew-normal on the estimated regression coefficients, with 95% credible intervals, in the slope model.

Parameter	*cVL*_*2*_ *adjusted model 1*		*cVL*_*2*_ *adjusted model 4*	
	Estimate	95% CI	Estimate	95% CI
**Female-sex**	23.8	(12.53, 34.96)	23.8	(12.61, 35.12)
**Baseline age**	-6.3	(-11.57, -1.08)	-6.4	(-11.61, -1.14)
**Baseline CD4 count**	82.9	(77.4, 88.38)	82.8	(77.36, 88.29)
**Baseline log viral load**	7.5	(2.03, 12.92)	7.5	(2.06, 12.91)
**Time on treatment**	55.7	(48.83, 62.65)	55.7	(48.72, 62.57)
**Time on treatment-squared**	-22.7	(-24.86, -20.78)	-22.8	(-24.84, -20.78)
**cumulative log viral load**	**-19.6**	**(-28.25, -10.93)**	**-19.4**	**(-28.12, -10.74)**

cVL_2_ –cumulative HIV log_10_ viral load. All estimates reported as posterior mean and 95% credible intervals.

### Using informative priors

This improved estimation of covariate effects in model 1^*^. For example, the mean annual effect of cVL_2_ on immune response increases from -19.6 cell/μL (95%CI: -28.25, -10.93) in *model 1*, i.e. without informative priors, to -19.7 cell/μL (95%CI: -28.35, -11.10) in model 1^*^, i.e. with informative priors. For additional details refer to Table E in S2 Appendix.

In the asymptote model, the distribution of the random-effects of the polynomial version is visibly skewed, Figure D in S2 Appendix, implying that the use of a Gaussian normal distribution for the random effects is inappropriate. Using a ***SN*** yields a significantly lower cDIC compared to a Gaussian distribution, *model 7* = 829 vs *model 6* = 1695 in the adjusted model and in the unadjusted, *model 7* = 1108 vs *model 6* = 1716, Table H in S2 Appendix. The adjusted ***SN*** model still has the lowest cDIC among all asymptote models. Overall, the ***SN*** model has slightly smaller CIs compared to the normal random effects model, Table 5. cVL_2_ remains a significant covariate in the asymptote model, Table 5.

**Table 5:** The effect of changing from a normal to a skew-normal distribution on the random-effects on the regression coefficients, with 95% credible intervals, in the asymptote model.

Parameter	*cVL*_*2*_ *adjusted model 6*		*cVL*_*2*_ *adjusted model 7*	
	Estimate	95% CI	Estimate	95% CI
**Female-sex**	6.35	(3.019, 14.382)	6.52	(3.004, 14.397)
**Baseline age**	0.54	(0.389, 0.740)	0.54	(0.386, 0.748)
**Baseline CD4 count**	3.00	(2.175, 4.145)	3.04	(2.235, 4.242)
**Baseline log viral load**	1.26	(0.905, 1.799)	1.27	(0.904, 1.785)
**Time on treatment**	4.08	(3.019, 5.579)	4.04	(3.034, 5.425)
**Time on treatment—squared**	0.65	(0.550 0.745)	0.65	(0.564, 0.746)
**cumulative log viral load**	**0.42**	**(0.236, 0.730)**	**0.41**	**(0.236, 0.718)**

cVL_2_ –cumulative HIV log_10_ viral load. All estimates reported as posterior odds ratios with 95% credible intervals (CI).

## Discussion

Proper adherence to ART, in the absence of HIV drug resistance, is sufficient to suppress HIV VL in plasma. When this is not achieved ongoing viral replication causes progressive depletion of CD4+ T-cells through direct and indirect mechanisms [8,29,39]. However, the extent of damage by a detectable VL in the short-term may not be readily apparent as CD4 counts are inherently variable within and between people [15–17].

Using prospectively defined models on an unpublished SSA HIV treatment cohort, we demonstrated that long-term consequences of a history of detectable VL, i.e. the cVL_2_ biomarker, on immune response while on ART. cVL_2_ is strongly associated with, and provides better model fits for, both slope and asymptote CD4 count models. These combined CD4 count-VL dynamics have not previously been demonstrated in causal statistical models and are in apparent agreement with the findings of biologically ‘mechanistic’ mathematical models [11]. After adjusting for sex, baseline age, baseline CD4 count, baseline log_10_ VL and time on treatment, each log_10_ copy-year/mL increase in cVL_2_ was associated with a decrease in slope of CD4 counts and lower odds of having a CD4 count of ≥500 cells/μL while on ART. The median probability of having a CD4 count ≥500 cells/μL was still < 0.5 among patients with a baseline CD4 count ≤100 cells/μL after 5 years of follow-up. Having a CD4 count ≥500 cells/μL while on ART was previously associated with a similar life expectancy to that in the normal population [21]. Our findings provide evidence for increased benefits of early ART initiation, as the probability of having a CD4 counts ≥500 cells/μL is then increased.

In addition to baseline log VL, increasing baseline age, associated with a reduction in the size and functionality of the thymus [5,29], and lower baseline CD4 counts [22,40,41] are associated with ineffective CD4 count recovery. Lawn et al. [42] have shown that late ART initiators, with baseline CD4 count <200 cells/ μL, equivalent to 94% of our cohort, often take longer than 5 years of treatment before they reach CD4 count ≥500 cells/μL. In our cohort and elsewhere [22], patients with baseline CD4 count ≤ 200 cells/μL *never* reached a 0.5 median predicted probability of having a CD4 count ≥500 cells/μL. Further, such patients are at risk of developing suboptimal immune response, i.e. failure to reach CD4 count >350 cells/μL after 5 years of ART [43]. Patients with >200 cells/μL at baseline experienced a decline in the median predicted probability of having CD4 count ≥500 cells/μL after 3 years, Fig 2A. It is possible that patients initiated on ART at >200 cells/μL quickly reach a CD4 count ceiling which, according to Williams et al 2006 and Kulkarni et al 2011 is related to pre-infection and immediate post-infection CD4 count levels [44,45]. Our findings of better CD4 count recovery in females compared to males is consistent with prior findings [8,22,25,29,41], even though we had fewer males than females a trend which is common in clinical settings in sub-Saharan Africa [46].

Results from randomized controlled trials such as the ‘Strategies for Management of Antiretroviral Therapy’ (SMART) [47], ‘Strategic Timing of Antiretroviral Therapy (START)’ [48] and ‘Trial of Early Antiretrovirals and Isoniazid Preventive Therapy in Africa’ (TEMPRANO) [49], indicated benefits of early initiation of ART to reduce AIDS and non-AIDS infection in HIV patients. Currently, the World Health Organization recommendations are to initiate ART in all newly diagnosed HIV patients, regardless of their CD4 count a diagnosis. However, many people living with HIV are still initiated late on ART, reflected by low baseline CD4 counts [50], possibly due to programmatic bottlenecks, e.g. drug stock-outs, limited clinic space, late diagnosis or patient decisions to defer ART. Our findings are relevant resource-limited settings in highly HIV burdened sub-Saharan Africa.

cVL_2_ attempts to capture cumulative HIV antigenic exposure due to detectable viral load (>400 copies/mL) during ART. However, cVL_2_ estimation improves with narrower sampling intervals [13,51]. Increasing VL sampling frequency in resource-limited settings is unrealistic as it is expensive. Mechanistic models have shown that the HIV virus has a short half-life of approximately 2 days [52], which implies that during ART, an HIV VL test shows the *current* VL rather than a time-averaged measure across the interval or up to a few weeks before the sampling visit. Considering this, interpreting VL tests may not be as informative as time-averaged cVL_2_, which may be a relevant biomarker for cumulative antigenic pressure.

Our models do not adjust for duration of HIV infection prior to ART initiation as this information was not available. This may have resulted in biased model estimates for cVL_2_. However, we did control for baseline CD4 count which, although perhaps not ideal due to inter-patient variability in the rate of CD4 decline prior to ART [53], is frequently used as a surrogate marker for duration of infection [54]. Further, approaches for adjusting for pre-ART VLs, as summarized in a prior study [13], may be of limited value as they are dependent on an individual’s innate viral controlling ability. In the future, adjusting similar models with data for HIV ‘recency’ assay results [55,56] if available, may serve as an additional indication for duration of infection.

A Bayesian approach was helpful in that it enabled us to apply informative prior estimates from historical models, although this was limited to only those that assume Gaussian normality [12,25,36]. Assuming normal distributions for random effects that are intrinsically not normally distributed is questionable and likely to produce biased results [57,58]. We found that models with both ***SN*** distributions for the random-effects and measurement errors produced substantially smaller cDIC’s, i.e. the best model fits. The predicted distributions for the random-effects in both slope and asymptote models were observably skew, as can be seen in Figure C in S2 Appendix and even more so in Figure D in S2 Appendix. Thus, using ***SN*** distributions is likely to have improved the description of the dataset as a whole. Having said that, the ***β***-parameters and 95%CIs obtained after using non-informative and informative priors were similar, likely owing to the relatively large size of the dataset [59].

The considered models all assumed that when patients have an incomplete follow-up that either: (1) the reason for incompleteness is not related to the outcome (Missing completely at random) or (2) could have been predicted from past history (missing at random). Of course, it can also be that the incompleteness is related in a more complicated manner to the outcome (missing not at random). However, there is no clear solution on how to deal with this case. Some possible approaches are joint modelling, sensitivity analyses assuming different mechanisms for the incompleteness, etc. Addressing missing not at random missingness mechanisms thus involve a lot of extra considerations. The possible impact of this missingness mechanism on our data will be the topic of future research.

This study has limitations. As for most Bayesian models based on sizeable datasets, running them was computationally intensive. Estimation of cVL_2_ may also have been affected by sampling frequency, as previously alluded to [13]. VL was measured bi-annually in the current cohort; thus, the effect of detectable VL might have excluded unmeasured viral rebounds occurring between sampling intervals. This cohort included patients who were initiated on ART at very low median CD4 counts, i.e. 87 cells/μL (IQR: 43, 144), and may consequently have been predisposed to poor CD4 count responses, Fig 1, as compared to cohorts with early ART initiation in resource-rich settings. Our results may be prone to a ‘survival’ bias as 213 patients, who were excluded from this study because they only had a baseline visit, were older and had a higher baseline VL.

Taken together, the results of this study show promising use of the cVL_2_ metric to describe CD4 count response in ART patients. Models were prospectively defined and tested on the data of an as yet undescribed SSA HIV treatment cohort. Bayesian methods enabled the incorporation of prior information from historical studies. All assumptions were explicitly defined facilitating reproducibility and standardization. In the future, quantifying the effect of viral rebounds occurring between sampling intervals and duration of HIV infection may help improve our understanding of the effect of cVL_2_.

## Supporting information

S1 Appendix WinBugs/OpenBugs code for all the models analyzed. (DOCX)

S2 Appendix Additional tables and figures. (DOCX)
