# Correction: Cumulative viral load as a predictor of CD4+ T-cell response to antiretroviral therapy using Bayesian statistical models

**DOI:** 10.1371/journal.pone.0228218

**Published:** 2020-01-16

**Authors:** Joseph B. Sempa, Theresa M. Rossouw, Emmanuel Lesaffre, Martin Nieuwoudt

There is an error in affiliation 2 for author Theresa M. Rossouw. The correct affiliation 2 is: Department of Immunology, University of Pretoria, Pretoria, South Africa.

The affiliation for the fourth author is incorrectly indicated as their current address. Martin Nieuwoudt is affiliated with: Institute for Biomedical Engineering (IBE), Mechanical and Mechatronic Engineering,

Stellenbosch University, Western Cape, South Africa.
