# Supplementary material for: Cumulative viral load as a predictor of CD4+ T-cell response to antiretroviral therapy using Bayesian statistical models
Source: PLoS One. 2019 Nov 13;14(11):e0224723. doi: 10.1371/journal.pone.0224723 (PMC6853324; doi:10.1371/journal.pone.0224723)
Supplement: S1 Appendix — (DOCX) [file pone.0224723.s001.docx]

**S1 Appendix:**

**Slope of CD4 count models:**

**Slope model 1: primary cumulative log viral load model**

model{

for( i in 1 : N ) {

for( j in 1 : M ) {

CD4[i , j] ~ dnorm(mu[i , j], tau.int)

mu[i , j] <- beta[1] + beta[2] * sex.f[i] + beta[3] * age_count[i] + beta[4] * base.cd4[i] + beta[5] * base.logvl[i] + beta[6] * (yrs.fu[j] - 2.5) + beta[7] * pow(yrs.fu[j] - 2.5, 2) + beta[8] * cumvl[i , j] * cumvlFlag[i , j] + U[i , 1] + U[i , 2] * yrs.fu[j]

}

U[i , 1:2] ~ dmnorm(U0[], tau[ , ])

basecd4.imp[i] <- cut(base.cd4[i])

baselogvl.imp[i] <- cut(base.logvl[i])

base.cd4[i] ~ dnorm(mu1[i], tau1.int)

mu1[i] <- beta1[1] + beta1[2] * sex.f[i] +

beta1[3] * age_count[i]

base.logvl[i] ~ dnorm(mu2[i], tau2.int)

mu2[i] <- beta2[1] + beta2[2] * sex.f[i] +

beta2[3] * age_count[i]

sex.f[i] ~ dbern(p[i])

p[i] <- max(1.0E-5, min(0.99999, ptemp[i]))

logit(ptemp[i]) <- beta3[1] +

beta3[2] * age_count[i] +

beta3[3] * baselogvl[i] + beta3[4] * basecd4[i]

baselogvl[i]~dnorm(mua[i],taua.int)

mua[i] <- betaa[1] + betaa[2]*sex.f[i] + betaa[3]*age.count[i]

basecd4[i]~dnorm(mub[i],taub.int)

mub[i] <- betab[1] +betab[2]*sex.f[i] +

betab[3]*age.count[i]

###End of missing data imputation section

for( j in 1 : M ) {

cd4.p1[i , j] ~ dnorm(mu[i , j], tau.int)

r12[i , j] <- CD4[i , j] - cd4.p1[i , j]

sqr[i , j] <- r12[i , j] * r12[i , j]

}

}

mspe <- mean(sqr[ , ])

sigmau0 ~ dunif(0, 100)

sigmau1 ~ dunif(0, 100)

cor ~ dunif(-1, 1)

sigmaU[1 , 1] <- pow(sigmau0, 2)

sigmaU[2 , 2] <- pow(sigmau1, 2)

sigmaU[1 , 2] <- sigmau0 * sigmau1 * cor

sigmaU[2 , 1] <- sigmaU[1 , 2]

tau[1:2 , 1:2] <- inverse(sigmaU[ , ])

sigma1 <- pow(tau.int,-2)

tau.int ~ dgamma(0.001,0.001)

sigma1.imp <- pow(tau1.int,-2)

tau1.int ~ dgamma(0.001,0.001)

sigma2.imp <- pow(tau2.int,-2)

tau2.int ~ dgamma(0.001,0.001)

sigmaa.imp <- pow(taua.int,-2)

taua.int ~ dgamma(0.001,0.001)

sigmab.imp <- pow(taub.int,-2)

taub.int ~ dgamma(0.001,0.001)

for( k in 1 : 8 ) {

beta[k] ~ dnorm(0, 0.001)

}

for( k in 1 : 3 ) {

beta1[k] ~ dnorm(0, 0.001)

}

for( k in 1 : 3 ) {

beta2[k] ~ dnorm(0, 0.001)

}

for( k in 1 : 4 ) {

beta3[k] ~ dnorm(0, 0.001)

}

for (k in 1:3) { ##imputations of baselogVL

betaa[k]~dnorm(0,0.001)

}

for (k in 1:3) { ##imputations of basecd4

betab[k]~dnorm(0,0.001)

}

}

**Model 1 priors:**

In this model, we used vague priors on all parameters as we wanted the prior to be roughly constant in the interval where the likelihood is not zero (18). In this section we use the variance and not precision notation, i.e. $x\sim N\left( mu, s^{2} \right)$ not $x\sim N\left( mu, \Psi\right)$ with the precision $\Psi=s^{-2}$, for all normally distributed priors even though we used precision notation in the OpenBUGS model (18). The following priors were implemented: For fixed effects vectors $\boldsymbol{\beta}$ for the main and $\boldsymbol{\phi}_{i}$ for $i=\left( 1,\ldots,5 \right)$ imputation sub models, we specified an independent Gaussian distribution i.e. $\boldsymbol{N}\left( 0, 1000 \right)$; scale parameter for CD4 count response $\sigma_{\varepsilon}$ had a conditional conjugate prior ‘*tau.int’* with an Inverse-gamma distribution, i.e. $\boldsymbol{IG}\left( 0.001, 0.001 \right)$ and the same distribution for scale parameters $\boldsymbol{\tau}_{\boldsymbol{\varepsilon}}=\left( \tau_{1\varepsilon}, \tau_{2\varepsilon}, \tau_{3\varepsilon}, \tau_{4\varepsilon} \right)$ denoted as ‘*tau1.int*’, ‘*tau2.int*’‘*taua.int*’ and ‘*taub.int*’, respectively, for each imputation model; for random-effects covariance matrix $\Sigma_{b}$ denoted as ‘*tau*’, we specified uniform distributions for the standard deviations $\sigma_{0}$ denoted *‘sigmau0’*and $\sigma_{1}$ denoted ‘*sigmau1*’ for random intercept and slope i.e. $\boldsymbol{U}\left( 0, 100 \right)$; and for the correlation $\rho$ denoted as *‘cor’* a Uniform distribution $\boldsymbol{U}\left( 1, -1 \right)$.

**Slope model with cumulative log viral load – model 2: cubic b-splines with 3 inner knots**

model{

for( i in 1 : N ) {

for( j in 1 : M ) {

CD4[i , j] ~ dnorm(mu[i , j], tau.int)

mu[i , j] <- beta[1] * sex.f[i] + beta[2] * age_count[i] + beta[3] * base.cd4[i] + beta[4] * base.logvl[i] + beta[5] * cumvl[i , j] * cumvlFlag[i , j] + U[i , 1] + U[i , 2] * yrs.fu[j] + bspline[1] * time1[i , j] + bspline[2] * time2[i , j] + bspline[3] * time3[i , j] + bspline[4] * time4[i , j] + bspline[5] * time5[i , j] + bspline[6] * time6[i , j] + bspline[7] * time7[i , j] + bspline[8] * time8[i , j]

}

U[i , 1:2] ~ dmnorm(U0[], tau[ , ])

basecd4.imp[i] <- cut(base.cd4[i])

baselogvl.imp[i] <- cut(base.logvl[i])

base.cd4[i] ~ dnorm(mu1[i], tau1.int)

mu1[i] <- beta1[1] + beta1[2] * sex.f[i] +

beta1[3] * age_count[i]

base.logvl[i] ~ dnorm(mu2[i], tau2.int)

mu2[i] <- beta2[1] + beta2[2] * sex.f[i] +

beta2[3] * age_count[i]

sex.f[i] ~ dbern(p[i])

p[i] <- max(1.0E-5, min(0.99999, ptemp[i]))

logit(ptemp[i]) <- beta3[1] + beta3[2] * age_count[i] +

beta3[3] * baselogvl[i] + beta3[4] * basecd4[i]

baselogvl[i]~dnorm(mua[i],taua.int)

mua[i] <- betaa[1] + betaa[2]*sex.f[i] + betaa[3]*age.count[i]

basecd4[i]~dnorm(mub[i],taub.int)

mub[i] <- betab[1] +betab[2]*sex.f[i] +

betab[3]*age.count[i]

###End of missing data imputation section

for( j in 1 : M ) {

cd4.p1[i , j] ~ dnorm(mu[i , j], tau.int)

r12[i , j] <- CD4[i , j] - cd4.p1[i , j]

sqr[i , j] <- r12[i , j] * r12[i , j]

}

}

mspe <- mean(sqr[ , ])

sigmau0 ~ dunif(0, 100)

sigmau1 ~ dunif(0, 100)

cor ~ dunif(-1, 1)

sigmaU[1 , 1] <- pow(sigmau0, 2)

sigmaU[2 , 2] <- pow(sigmau1, 2)

sigmaU[1 , 2] <- sigmau0 * sigmau1 * cor

sigmaU[2 , 1] <- sigmaU[1 , 2]

tau[1:2 , 1:2] <- inverse(sigmaU[ , ])

sigma1 <- pow(tau.int,-2)

tau.int ~ dgamma(0.001,0.001)

sigma1.imp <- pow(tau1.int,-2)

tau1.int ~ dgamma(0.001,0.001)

sigma2.imp <- pow(tau2.int,-2)

tau2.int ~ dgamma(0.001,0.001)

sigmaa.imp <- pow(taua.int,-2)

taua.int ~ dgamma(0.001,0.001)

sigmab.imp <- pow(taub.int,-2)

taub.int ~ dgamma(0.001,0.001)

for( k in 1 : 5 ) {

beta[k] ~ dnorm(0, 0.001)

}

for( k in 1 : 3 ) {

beta1[k] ~ dnorm(0, 0.001)

}

for( k in 1 : 3 ) {

beta2[k] ~ dnorm(0, 0.001)

}

for( k in 1 : 4 ) {

beta3[k] ~ dnorm(0, 0.001)

}

for( d in 1 : 8 ) {

bspline[d] ~ dnorm(0, 1.0E-6)

}

for (k in 1:3) { ##imputations of baselogVL

betaa[k]~dnorm(0,0.001)

}

for (k in 1:3) { ##imputations of basecd4

betab[k]~dnorm(0,0.001)

}

}

**Model 2 priors**

In this model, we used vague priors on all parameters as we wanted the prior to be roughly constant in the interval where the likelihood is not zero (18). In this section we use the variance and not precision notation, i.e. $x\sim N\left( mu, s^{2} \right)$ not $x\sim N\left( mu, \Psi\right)$ with the precision $\Psi=s^{-2}$, for all normally distributed priors even though we used precision notation in the OpenBUGS model (18). The following priors were implemented: Fixed effects vector $\boldsymbol{\beta}$ for the main and $\boldsymbol{\phi}_{\boldsymbol{i}}$ for $i=\left( 1,\ldots,5 \right)$ imputation sub models had an independent Gaussian distribution i.e. $\boldsymbol{N}\left( 0,1000 \right)$; spline terms $\boldsymbol{\theta}$ denoted ‘*bspline*’ were also assumed to have an independent Gaussian distribution $\boldsymbol{N}\left( 0, {10}^{6} \right)$ because this provides a small precision and would vary less compared to fixed effects; scale parameter for CD4 count response $\sigma_{\varepsilon}$ had a conditional conjugate prior ‘*tau.int’* with an Inverse-gamma distribution, i.e. $\boldsymbol{IG}\left( 0.001, 0.001 \right)$ and the same distribution for scale parameters $\boldsymbol{\tau}_{\boldsymbol{\varepsilon}}=\left( \tau_{1\varepsilon}, \tau_{2\varepsilon}, \tau_{3\varepsilon}, \tau_{4\varepsilon} \right)$ denoted as ‘*tau1.int*’, ‘*tau2.int*’‘*taua.int*’ and ‘*taub.int*’, respectively, for each imputation model; for random-effects covariance matrix $\Sigma_{b}$ denoted as ‘*tau*’, we specified uniform distributions for the standard deviations $\sigma_{0}$ denoted‘*sigmau0*’ and $\sigma_{1}$ denoted‘*sigmau1*’ for random intercept and slope i.e. $\boldsymbol{U}\left( 0, 100 \right)$; and for the correlation $\rho$ denoted as *‘cor’* a Uniform distribution $\boldsymbol{U}\left( 1, -1 \right)$ .

**Slope model with cumulative log viral load – Model 3: cubic B-splines with 5 inner knots**

model{

for( i in 1 : N ) {

for( j in 1 : M ) {

CD4[i , j] ~ dnorm(mu[i , j], tau.int)

mu[i , j] <- beta[1] * sex.f[i] + beta[2] * age_count[i] + beta[3] * base.cd4[i] + beta[4] * base.logvl[i] + beta[5] * cumvl[i , j] * cumvlFlag[i , j] + U[i , 1] + U[i , 2] * yrs.fu[j] + bspline[1] * time1[i , j] + bspline[2] * time2[i , j] + bspline[3] * time3[i , j] + bspline[4] * time4[i , j] + bspline[5] * time5[i , j] + bspline[6] * time6[i , j] + bspline[7] * time7[i , j] + bspline[8] * time8[i , j] + bspline[9] * time9[i , j] + bspline[10] * time10[i , j]

}

U[i , 1:2] ~ dmnorm(U0[], tau[ , ])

basecd4.imp[i] <- cut(base.cd4[i])

baselogvl.imp[i] <- cut(base.logvl[i])

base.cd4[i] ~ dnorm(mu1[i], tau1.int)

mu1[i] <- beta1[1] + beta1[2] * sex.f[i] +

beta1[3] * age_count[i]

base.logvl[i] ~ dnorm(mu2[i], tau2.int)

mu2[i] <- beta2[1] + beta2[2] * sex.f[i] +

beta2[3] * age_count[i]

sex.f[i] ~ dbern(p[i])

p[i] <- max(1.0E-5, min(0.99999, ptemp[i]))

logit(ptemp[i]) <- beta3[1] +

beta3[2] * age_count[i] +

beta3[3] * baselogvl[i] + beta3[4] * basecd4[i]

baselogvl[i]~dnorm(mua[i],taua.int)

mua[i] <- betaa[1] + betaa[2]*sex.f[i] + betaa[3]*age.count[i]

basecd4[i]~dnorm(mub[i],taub.int)

mub[i] <- betab[1] +betab[2]*sex.f[i] +

betab[3]*age.count[i]

###End of missing data imputation section

for( j in 1 : M ) {

cd4.p1[i , j] ~ dnorm(mu[i , j], tau.int)

r12[i , j] <- CD4[i , j] - cd4.p1[i , j]

sqr[i , j] <- r12[i , j] * r12[i , j]

}

}

mspe <- mean(sqr[ , ])

sigmau0 ~ dunif(0, 100)

sigmau1 ~ dunif(0, 100)

cor ~ dunif(-1, 1)

sigmaU[1 , 1] <- pow(sigmau0, 2)

sigmaU[2 , 2] <- pow(sigmau1, 2)

sigmaU[1 , 2] <- sigmau0 * sigmau1 * cor

sigmaU[2 , 1] <- sigmaU[1 , 2]

tau[1:2 , 1:2] <- inverse(sigmaU[ , ])

sigma1 <- pow(tau.int,-2)

tau.int ~ dgamma(0.001,0.001)

sigma1.imp <- pow(tau1.int,-2)

tau1.int ~ dgamma(0.001,0.001)

sigma2.imp <- pow(tau2.int,-2)

tau2.int ~ dgamma(0.001,0.001)

sigmaa.imp <- pow(taua.int,-2)

taua.int ~ dgamma(0.001,0.001)

sigmab.imp <- pow(taub.int,-2)

taub.int ~ dgamma(0.001,0.001)

for( k in 1 : 5 ) {

beta[k] ~ dnorm(0, 0.001)

}

for( k in 1 : 3 ) {

beta1[k] ~ dnorm(0, 0.001)

}

for( k in 1 : 3 ) {

beta2[k] ~ dnorm(0, 0.001)

}

for( k in 1 : 4 ) {

beta3[k] ~ dnorm(0, 0.001)

}

for( d in 1 : 10 ) {

bspline[d] ~ dnorm(0, 1.0E-6)

}

for (k in 1:3) { ##imputations of baselogVL

betaa[k]~dnorm(0,0.001)

}

for (k in 1:3) { ##imputations of basecd4

betab[k]~dnorm(0,0.001)

}

}

**Model 3 priors**

In this model, we used vague priors on all parameters as we wanted the prior to be roughly constant in the interval where the likelihood is not zero (18). In this section we use the variance and not precision notation, i.e. $x\sim N\left( mu, s^{2} \right)$ not $x\sim N\left( mu, \Psi\right)$ with the precision $\Psi=s^{-2}$, for all normally distributed priors even though we used precision notation in the OpenBUGS model (18). The following priors were implemented: fixed effects vector $\boldsymbol{\beta}$ for the main and $\boldsymbol{\phi}_{\boldsymbol{i}}$ for $i=\left( 1,\ldots,5 \right)$ imputation sub models had an independent Gaussian distribution i.e. $\boldsymbol{N}\left( 0,1000 \right)$; spline terms $\boldsymbol{\theta}$ denoted ‘*bspline*’ were also assumed to have an independent Gaussian distribution, i.e. $\boldsymbol{N}\left( 0, {10}^{6} \right)$ because this provides a small precision and would vary less compared to fixed effects; scale parameter for CD4 count response $\sigma_{\varepsilon}$ had a conditional conjugate prior ‘*tau.int’* with an Inverse-gamma distribution, i.e. $\boldsymbol{IG}\left( 0.001, 0.001 \right)$ and the same distribution for scale parameters $\boldsymbol{\tau}_{\boldsymbol{\varepsilon}}=\left( \tau_{1\varepsilon}, \tau_{2\varepsilon}, \tau_{3\varepsilon}, \tau_{4\varepsilon} \right)$ denoted as ‘*tau1.int*’, ‘*tau2.int*’‘*taua.int*’ and ‘*taub.int*’, respectively, for each imputation model; for random-effects covariance matrix $\Sigma_{b}$ denoted as ‘*tau*’, we specified uniform distributions for the standard deviations $\sigma_{0}$ denoted ‘*sigmau0*’ and $\sigma_{1}$ denoted ‘*sigmau1*’ for random intercept and slope i.e. $\boldsymbol{U}\left( 0, 100 \right)$; and for the correlation $\rho$ denoted as *‘cor’* a Uniform distribution $\boldsymbol{U}\left( 1, -1 \right)$.

**Slope model with cumulative log viral load – Model 4: Skew-normal random-effects**

model{

for( i in 1 : N ) {

for( j in 1 : M ) {

CD4[i , j] ~ dnorm(mu[i , j], tau.int)

mu[i , j] <- beta[1] + beta[2] * sex.f[i] + beta[3] * age_count[i] + beta[4] * base.cd4[i] + beta[5] * base.logvl[i] + beta[6] * (yrs.fu[j] - 2.5) + beta[7] * pow(yrs.fu[j] - 2.5, 2) + beta[8] * cumvl[i , j] * cumvlFlag[i , j] + U[i , 1] + U[i , 2] * yrs.fu[j]

}

U0.t[i , 1] ~ dnorm(0, 1)

U0.t[i , 2] ~ dnorm(0, 1)

U0[i , 1] <- deltab.abs[1] * U0.t[i , 1]

U0[i , 2] <- deltab.abs[2] * U0.t[i , 2]

U1[i , 1:2] ~ dmnorm(meanb[1:2], tau[1:2 , 1:2])

for( ii in 1 : 2 ) {

U[i , ii] <- U0[i , ii] + U1[i , ii]

}

basecd4.imp[i] <- cut(base.cd4[i])

baselogvl.imp[i] <- cut(base.logvl[i])

base.cd4[i] ~ dnorm(mu1[i], tau1.int)

mu1[i] <- beta1[1] + beta1[2] * sex.f[i] +

beta1[3] * age_count[i]

base.logvl[i] ~ dnorm(mu2[i], tau2.int)

mu2[i] <- beta2[1] + beta2[2] * sex.f[i] +

beta2[3] * age_count[i]

sex.f[i] ~ dbern(p[i])

p[i] <- max(1.0E-5, min(0.99999, ptemp[i]))

logit(ptemp[i]) <- beta3[1] +

beta3[2] * age_count[i] +

beta3[3] * baselogvl[i] + beta3[4] * basecd4[i]

baselogvl[i]~dnorm(mua[i],taua.int)

mua[i] <- betaa[1] + betaa[2]*sex.f[i] + betaa[3]*age.count[i]

basecd4[i]~dnorm(mub[i],taub.int)

mub[i] <- betab[1] +betab[2]*sex.f[i] +

betab[3]*age.count[i]

###End of missing data imputation section

for( j in 1 : M ) {

cd4.p1[i , j] ~ dnorm(mu[i , j], tau.int)

r12[i , j] <- CD4[i , j] - cd4.p1[i , j]

sqr[i , j] <- r12[i , j] * r12[i , j]

}

}

mspe <- mean(sqr[ , ])

Om[1 , 1] <- a * Omega[1 , 1]

Om[1 , 2] <- a * Omega[1 , 2]

Om[2 , 1] <- a * Omega[2 , 1]

Om[2 , 2] <- a * Omega[2 , 2]

Q[1:2 , 1:2] ~ dwish(Om[1:2 , 1:2], 3)

tau[1 , 1] <- pow(D1, -2) * Q[1 , 1]

tau[1 , 2] <- pow(D1, -1) * pow(D2, -1) * Q[1 , 2]

tau[2 , 1] <- pow(D2, -1) * pow(D1, -1) * Q[2 , 1]

tau[2 , 2] <- pow(D2, -2) * Q[2 , 2]

sigmab2[1:2, 1:2] <- inverse(tau[ , ])

sigmab[1] <- sqrt(sigmab2[1 , 1])

sigmab[2] <- sqrt(sigmab2[2 , 2])

corrb <- sigmab2[1 , 2] / sigmab[1] * sigmab[2]

D1 ~ dunif(0, 100)

D2 ~ dunif(0, 100)

deltab[1] ~ dnorm(0.0, 1.0E-6)

deltab[2] ~ dnorm(0.0, 1.0E-6)

deltab.abs[1] <- abs(deltab[1])

deltab.abs[2] <- abs(deltab[2])

sigma1 <- pow(tau.int,-2)

tau.int ~ dgamma(0.001,0.001)

sigma1.imp <- pow(tau1.int,-2)

tau1.int ~ dgamma(0.001,0.001)

sigma2.imp <- pow(tau2.int,-2)

tau2.int ~ dgamma(0.001,0.001)

sigmaa.imp <- pow(taua.int,-2)

taua.int ~ dgamma(0.001,0.001)

sigmab.imp <- pow(taub.int,-2)

taub.int ~ dgamma(0.001,0.001)

for( k in 1 : 8 ) {

beta[k] ~ dnorm(0, 0.001)

}

for( k in 1 : 3 ) {

beta1[k] ~ dnorm(0, 0.001)

}

for( k in 1 : 3 ) {

beta2[k] ~ dnorm(0, 0.001)

}

for( k in 1 : 4 ) {

beta3[k] ~ dnorm(0, 0.001)

}

for (k in 1:3) { ##imputations of baselogVL

betaa[k]~dnorm(0,0.001)

}

for (k in 1:3) { ##imputations of basecd4

betab[k]~dnorm(0,0.001)

}

}

**Model 4 priors**

In this model, we used vague priors on all parameters as we wanted the prior to be roughly constant in the interval where the likelihood is not zero (18). In this section we use the variance and not precision notation, i.e. $x\sim N\left( mu, s^{2} \right)$ not $x\sim N\left( mu, \Psi\right)$ with the precision $\Psi=s^{-2}$, for all normally distributed priors even though we used precision notation in the OpenBUGS model (18). The following priors were implemented: fixed effects vector $\boldsymbol{\beta}$ for the main and $\boldsymbol{\phi}_{\boldsymbol{i}}$ for $i=\left( 1,\ldots,5 \right)$ imputation sub models had an independent Gaussian distribution i.e. $\boldsymbol{N}\left( 0,1000 \right)$; the scale parameter for CD4 count response $\sigma_{\varepsilon}$, had a conditional conjugate prior ‘*tau.int’* with an Inverse-gamma distribution, i.e. $\boldsymbol{IG}\left( 0.001, 0.001 \right)$ and the same distribution for scale parameters $\boldsymbol{\tau}_{\boldsymbol{\varepsilon}}= \left( \tau_{1\varepsilon}, \tau_{2\varepsilon}, \tau_{3\varepsilon}, \tau_{4\varepsilon} \right)$ denoted as ‘*tau1.int*’, ‘*tau2.int*’‘*taua.int*’ and ‘*taub.int*’, respectively for each imputation model. The coefficients of the random effects $\boldsymbol{\alpha}=\left( \alpha_{i,1},\alpha_{i,2} \right)$ for each i^th^ patient denoted $U_{i,1}$and $U_{i,2}$ for intercept and slope, respectively, were multivariate normally distributed and had a dispersion parameter denoted ‘*U0*’ for each intercept and slope, which is a product of a normally distributed i.e. $\boldsymbol{N}\left( 0, {10}^{6} \right)$ absolute parameter $\boldsymbol{\Delta}_{\boldsymbol{b}}=\left( \Delta_{1b}, \Delta_{2b} \right)$ denoted as ‘*deltab.abs*’, and the respective $\boldsymbol{\vartheta}_{b}=\left( \vartheta_{1b}, \vartheta_{2b} \right)$ denoted ‘*U0.t*’—absolute values from a normal distribution $\boldsymbol{N}\left( 0, 1 \right)$. The product makes a distribution of ‘*U0*’ peaked around zero, which forms a favourable distribution for random-effects. The random-effects covariance matrix $\Sigma_{b}$ denoted as *‘tau’* follows an Inverse-Wishart distribution, where Wishart distribution i.e. $\boldsymbol{W}\left( R, 3 \right)$ ($R$ is a 2-by-2 diagonal matrix of 0.1) is multiplied by uniformly distributed i.e. $\boldsymbol{U}\left( 0, 100 \right)$ shape parameters $\boldsymbol{\gamma}_{\boldsymbol{b}}\boldsymbol{=}\left( \gamma_{1b}, \gamma_{2b} \right)$ denoted as ‘*D1*’ and *‘D2’* for intercept and slope, respectively.

**Slope model with cumulative log viral load – Model 5: Skew-normal measurement error and random-effects**

model {

for(i in 1:N){

for (j in 1:M) {

c[i,j] ~ dnorm(0,1)

w[i,j] <- abs(c[i,j])

CD4[i,j] ~ dnorm(mu[i,j], tau.int)

mu[i,j] <- beta[1] +

beta[2]*sex.f[i] +

beta[3]*age_count[i]+

beta[4]*base.cd4[i]+

beta[5]*base.logvl[i] +

beta[6]*(yrs.fu[j]-2.5)+

beta[7]*pow((yrs.fu[j]-2.5),2)+

beta[8]*(cumvl[i,j]*cumvlFlag[i,j])+

alpha[1]*U[i,1] +

alpha[2]*U[i,2]*(yrs.fu[j]-2.5) +

delta.abs*w[i,j]

}

for(k in 1:2){

ub[i,k] ~ dnorm(0,1)

wb[i,k] <- abs(ub[i,k])

ab[i,k] <- deltab.abs[k]*wb[i,k]

}

U[i,1:2] ~ dmnorm(ab[i,1:2], taub[1:2,1:2])

##Missing data imputation section for 59 patients

basecd4.imp[i] <- cut(base.cd4[i])

baselogvl.imp[i] <- cut(base.logvl[i])

#imputation model for baseline CD4

base.cd4[i]~dnorm(mu1[i],tau1.int)

mu1[i] <- beta1[1] +

beta1[2]*sex.f[i] +

beta1[3]*age_count[i]

#imputation model for baseline log VL

base.logvl[i]~dnorm(mu2[i],tau2.int)

mu2[i] <- beta2[1] +

beta2[2]*sex.f[i] +

beta2[3]*age_count[i]

#imputation model for gender

sex.f[i] ~ dbern(p[i])

p[i]<-max(0.00001,min(0.99999, ptemp[i]))

logit(ptemp[i]) <- beta3[1] +

beta3[2]*age_count[i]+

beta3[3]*baselogvl[i]+

beta3[4]*basecd4[i]

baselogvl[i]~dnorm(mua[i],taua.int)

mua[i] <- betaa[1] + betaa[2]*sex.f[i] + betaa[3]*age.count[i]

basecd4[i]~dnorm(mub[i],taub.int)

mub[i] <- betab[1] +betab[2]*sex.f[i] +

betab[3]*age.count[i]

###End of missing data imputation section

for (j in 1: M) {

cd4.p1[i,j] ~ dnorm(mu[i, j], tau.int)

r12[i,j] <- CD4[i,j]-cd4.p1[i,j]

sqr[i,j] <- r12[i,j]*r12[i,j]

}

}

mspe<-mean(sqr[,])

##Changing the cov.-matrix of the Wishart from identity to a multiplication of ##the identity

Om[1,1]<-a*Omega[1,1]

Om[1,2]<-a*Omega[1,2]

Om[2,1]<-a*Omega[2,1]

Om[2,2]<-a*Omega[2,2]

taub[1:2,1:2]~dwish(Om[1:2,1:2],3)

Q[1:2,1:2]<-inverse(taub[,])

sigmab2[1,1] <- Q[1,1]*pow(alpha[1],2)

sigmab2[1,2] <- Q[1,1]*alpha[1]*alpha[2]

sigmab2[2,1] <- Q[1,1]*alpha[1]*alpha[2]

sigmab2[2,2] <- Q[1,1]*pow(alpha[2],2)

for(l in 1:2){

sigmabb[l] <- sqrt(sigmab2[l,l])

}

corrb<-sigmab2[1,2]/(sigmabb[1]*sigmabb[2])

for(ll in 1:2){alpha[ll] ~dnorm(0.0,1.0E-6)}

deltab[1] ~ dnorm(0.0,1.0E-6)

deltab[2] ~ dnorm(0.0,1.0E-6)

deltab.abs[1] <- abs(deltab[1])

deltab.abs[2] <- abs(deltab[2])

delta~dnorm(0,1.0E-6)

delta.abs<-abs(delta)

sigma1 <- pow(tau.int,-2)

tau.int ~ dgamma(0.001,0.001)

sigma1.imp <- pow(tau1.int,-2)

tau1.int ~ dgamma(0.001,0.001)

sigma2.imp <- pow(tau2.int,-2)

tau2.int ~ dgamma(0.001,0.001)

sigmaa.imp <- pow(taua.int,-2)

taua.int ~ dgamma(0.001,0.001)

sigmab.imp <- pow(taub.int,-2)

taub.int ~ dgamma(0.001,0.001)

##Vage normal priors on the betas

for (k in 1:8) {

beta[k]~dnorm(0,0.001)

}

for (k in 1:3) { #imputations of base.CD4

beta1[k]~dnorm(0,0.001)

}

for (k in 1:3) { ##imputations of base.logVL

beta2[k]~dnorm(0,0.001)

}

for (k in 1:4) { ##imputations of sex

beta3[k]~dnorm(0,0.001)

}

for (k in 1:3) { ##imputations of baselogVL

betaa[k]~dnorm(0,0.001)

}

for (k in 1:3) { ##imputations of basecd4

betab[k]~dnorm(0,0.001)

}

}

**Model 5 priors**

In this model, we used vague priors on all parameters as we wanted the prior to be roughly constant in the interval where the likelihood is not zero (18). In this section we use the variance and not precision notation, i.e. $x\sim N\left( mu, s^{2} \right)$ not $x\sim N\left( mu, \Psi\right)$ with the precision $\Psi=s^{-2}$, for all normally distributed priors even though we used precision notation in the OpenBUGS model (18). The following priors were implemented: fixed effects vector $\boldsymbol{\beta}$ for the main and $\boldsymbol{\phi}_{\boldsymbol{i}}$ for $i=\left( 1,\ldots,5 \right)$ imputation sub models had an independent Gaussian distribution i.e. $\boldsymbol{N}\left( 0,1000 \right)$; the scale parameter for CD4 count response $\sigma_{\varepsilon}$ had a conditional conjugate prior ‘*tau.int’* with an Inverse-gamma distribution, i.e. $\boldsymbol{IG}\left( 0.001, 0.001 \right)$ and likewise the scale parameters $\boldsymbol{\tau}_{\boldsymbol{\varepsilon}}= \left( \tau_{1\varepsilon}, \tau_{2\varepsilon}, \tau_{3\varepsilon}, \tau_{4\varepsilon} \right)$ denoted as ‘*tau1.int*’, ‘*tau2.int*’‘*taua.int*’ and ‘*taub.int*’, respectively, for each imputation model. Random effects with normally distributed coefficient $\boldsymbol{\alpha}$ denoted ‘*alpha*’, $\boldsymbol{N}\left( 0, {10}^{6} \right)$. The mean for the multivariate normally distributed random effects ‘*ab*’ was estimated from the product of the dispersion parameters $\boldsymbol{\Delta}_{\boldsymbol{b}}=\left( \Delta_{1b}, \Delta_{2b} \right)$ denoted as *‘deltab.abs’*—absolute values from a normal distribution $\boldsymbol{N}\left( 0, {10}^{6} \right)$—and the respective $\boldsymbol{\vartheta}_{b}=\left( \vartheta_{1b}, \vartheta_{2b} \right)$ denoted ‘*wb*’—absolute values from a normal distribution $\boldsymbol{N}\left( 0, 1 \right)$. The product makes a distribution of *‘ab’* peaked, which forms a favourable multivariate distribution for the mean of the random-effects. The random-effects covariance matrix $\Sigma_{b}$ denoted *‘taub’* follows Inverse-Wishart distribution obtained from the product of a Wishart distribution $\boldsymbol{W}\left( R, 3 \right)$ ($R$ is a 2-by-2 matrix is a diagonal matrix of 0.1). The measurement error $\sigma_{\varepsilon}^{2}$ denoted as ‘*w*’ is always positive and is normally distributed such that $\boldsymbol{N}\left( 0, 1 \right)$. Further, we assumed a normal distribution $\boldsymbol{N}\left( 0, {10}^{6} \right)$ for the dispersion parameter for measurement error $\Delta_{\varepsilon_{i}}$ denoted ‘*delta.abs*’.

**Asymptote models:**

**Asymptote model with cumulative log viral load – Model 6: primary model**

model{

for( i in 1 : N ) {

for( j in 1 : M ) {

response[i , j] ~ dbern(p[i , j])

p[i , j] <- max(1.0E-5, min(0.99999, ptemp[i , j]))

logit(ptemp[i , j]) <- beta[1] + beta[2] * sex.f[i] + beta[3] * age.count[i] + beta[4] * base.cd4[i] + beta[5] * base.logvl[i] + beta[6] * (yrs.fu[j] - 2.5) + beta[7] * pow(yrs.fu[j] - 2.5, 2) + beta[8] * cumvl[i , j] * cumvlflag[i , j] + b[id[i] , 1] + b[id[i] , 2] * yrs.fu[j]

}

b[i , 1:2] ~ dmnorm(meanb[], tau[ , ])

basecd4.imp[i] <- cut(base.cd4[i])

baselogvl.imp[i] <- cut(base.logvl[i])

base.cd4[i] ~ dnorm(mu1[i], tau1.int)

mu1[i] <- beta1[1] + beta1[2] * sex.f[i] +

beta1[3] * age.count[i]

base.logvl[i] ~ dnorm(mu2[i], tau2.int)

mu2[i] <- beta2[1] + beta2[2] * sex.f[i] +

beta2[3] * age.count[i]

sex.f[i] ~ dbern(phi[i])

phi[i] <- max(1.0E-5, min(0.99999, phitemp[i]))

logit(phitemp[i]) <- beta3[1] +

beta3[2] * age.count[i] + beta3[3] * baselogvl[i] +

beta3[4] * basecd4[i]

}

sigmau0 ~ dunif(0, 100)

sigmau1 ~ dunif(0, 100)

cor ~ dunif(-1, 1)

sigmaU[1 , 1] <- pow(sigmau0, 2)

sigmaU[2 , 2] <- pow(sigmau1, 2)

sigmaU[1 , 2] <- sigmau0 * sigmau1 * cor

sigmaU[2 , 1] <- sigmaU[1 , 2]

tau[1:2 , 1:2] <- inverse(sigmaU[ , ])

sigma1 <- pow(tau.int,-2)

tau.int ~ dgamma(0.001,0.001)

sigma1.imp <- pow(tau1.int,-2)

tau1.int ~ dgamma(0.001,0.001)

sigma2.imp <- pow(tau2.int,-2)

tau2.int ~ dgamma(0.001,0.001)

sigmaa.imp <- pow(taua.int,-2)

taua.int ~ dgamma(0.001,0.001)

sigmab.imp <- pow(taub.int,-2)

taub.int ~ dgamma(0.001,0.001)

for( k in 1 : 8 ) {

beta[k] ~ dnorm(0, 0.001)

}

for( k in 1 : 3 ) {

beta1[k] ~ dnorm(0, 0.001)

}

for( k in 1 : 3 ) {

beta2[k] ~ dnorm(0, 0.001)

}

for( k in 1 : 4 ) {

beta3[k] ~ dnorm(0, 0.001)

}

for (k in 1:3) { ##imputations of baselogVL

betaa[k]~dnorm(0,0.001)

}

for (k in 1:3) { ##imputations of basecd4

betab[k]~dnorm(0,0.001)

}

}

**Model 6 priors**

In this model, we used vague priors on all parameters as we wanted the prior to be roughly constant in the interval where the likelihood is not zero (18). In this section we use the variance and not precision notation, i.e. $x\sim N\left( mu, s^{2} \right)$ not $x\sim N\left( mu, \Psi\right)$ with the precision $\Psi=s^{-2}$, for all normally distributed priors even though we used precision notation in the OpenBUGS model (18). The following priors were implemented: fixed effects vector $\boldsymbol{\beta}$ for the main and $\boldsymbol{\phi}_{i}$ for $i=\left( 1,\ldots,5 \right)$ imputation sub models had an independent Gaussian distribution i.e. $\boldsymbol{N}\left( 0,1000 \right)$; the scale parameters of the imputation sub models $\boldsymbol{\tau}_{\boldsymbol{\varepsilon}}= \left( \tau_{1\varepsilon}, \tau_{2\varepsilon}, \tau_{3\varepsilon}, \tau_{4\varepsilon} \right)$ denoted as ‘*tau1.int*’, ‘*tau2.int*’‘*taua.int*’ and ‘*taub.int*’, respectively, had conditional conjugate priors with an Inverse-gamma distribution, i.e. $\boldsymbol{IG}\left( 0.001, 0.001 \right)$; for random-effects covariance matrix $\Sigma_{b}$ denoted as ‘*tau*’, we specified uniform distributions for the standard deviations $\sigma_{0}$ denoted ‘*sigmau0*’ and $\sigma_{1}$ denoted ‘*sigmau1*’ for random intercept and slope i.e. $\boldsymbol{U}\left( 0, 100 \right)$; and for the correlation $\rho$ denoted as *‘cor’* a Uniform distribution $\boldsymbol{U}\left( 1, -1 \right)$.

**Asymptote model with cumulative log viral load – Model 7: skew-normal random-effects distribution model**

model

{

for(i in 1:N) {

for(j in 1:M) {

response[i,j] ~ dbern(p[i,j])

p[i,j]<-max(0.00001,min(0.99999, ptemp[i,j]))

logit(ptemp[i,j]) <- beta[1] + beta[2]*sex.f[i]+ beta[3]*age.count[i]+beta[4]*base.cd4[i] +

beta[5]*base.logvl[i]+

beta[6]*(yrs.fu[j]-2.5)+

beta[7]*pow((yrs.fu[j]-2.5),2)+

beta[8]*(cumvl[i,j]*cumvlflag[i,j])+

U[id[i],1]+ U[id[i],2]*yrs.fu[j]

}

U0.t[i,1] ~ dnorm(0,1)

U0.t[i,2] ~ dnorm(0,1)

U0[i,1] <- deltab.abs[1]*U0.t[i,1]

U0[i,2] <- deltab.abs[2]*U0.t[i,2]

U1[i,1:2] ~dmnorm(meanb[1:2],tau[1:2,1:2])

for(ii in 1:2){

U[i,ii]<- U0[i,ii] + U1[i,ii]

}

##Missing data imputation section for 59 patients

basecd4.imp[i] <- cut(base.cd4[i])

baselogvl.imp[i] <- cut(base.logvl[i])

#imputation model for baseline CD4

base.cd4[i]~dnorm(mu1[i],tau1.int)

mu1[i] <- beta1[1] +

beta1[2]*sex.f[i] +

beta1[3]*age_count[i]

#imputation model for baseline log VL

base.logvl[i]~dnorm(mu2[i],tau2.int)

mu2[i] <- beta2[1] +

beta2[2]*sex.f[i] +

beta2[3]*age_count[i]

#imputation model for gender

sex.f[i] ~ dbern(phi[i])

phi[i]<-max(0.00001,min(0.99999, phitemp[i]))

logit(phitemp[i]) <- beta3[1] +

beta3[2]*age_count[i]+

beta3[3]*baselogvl[i]+

beta3[4]*basecd4[i]

###End of missing data imputation section

}

##Changing the cov.-matrix of the Wishart from identity to a multiplication of the ##identity

Om[1,1]<-a*Omega[1,1]

Om[1,2]<-a*Omega[1,2]

Om[2,1]<-a*Omega[2,1]

Om[2,2]<-a*Omega[2,2]

Q[1:2,1:2]~dwish(Om[1:2,1:2],3)

tau[1,1]<-pow(D1,-2)*Q[1,1]

tau[1,2]<-pow(D1,-1)*pow(D2,-1)*Q[1,2]

tau[2,1]<-pow(D2,-1)*pow(D1,-1)*Q[2,1]

tau[2,2]<-pow(D2,-2)*Q[2,2]

sigmab2[1:2,1:2]<-inverse(tau[,])

sigmab[1]<-sqrt(sigmab2[1,1])

sigmab[2]<-sqrt(sigmab2[2,2])

corrb<-sigmab2[1,2]/(sigmab[1]*sigmab[2])

#####Priors for the scale parameters

D1~dunif(0,100)

D2~dunif(0,100)

deltab[1] ~ dnorm(0.0,1.0E-6)

deltab[2] ~ dnorm(0.0,1.0E-6)

deltab.abs[1] <- abs(deltab[1])

deltab.abs[2] <- abs(deltab[2])

sigma1 <- pow(tau.int,-2)

tau.int ~ dgamma(0.001,0.001)

sigma1.imp <- pow(tau1.int,-2)

tau1.int ~ dgamma(0.001,0.001)

sigma2.imp <- pow(tau2.int,-2)

tau2.int ~ dgamma(0.001,0.001)

sigmaa.imp <- pow(taua.int,-2)

taua.int ~ dgamma(0.001,0.001)

sigmab.imp <- pow(taub.int,-2)

taub.int ~ dgamma(0.001,0.001)

##Vage normal priors on the betas

for (k in 1:8) {

beta[k]~dnorm(0,0.001)

}

for (k in 1:3) { #imputations of base.CD4

beta1[k]~dnorm(0,0.001)

}

for (k in 1:3) { ##imputations of base.logVL

beta2[k]~dnorm(0,0.001)

}

for (k in 1:4) { ##imputations of sex

beta3[k]~dnorm(0,0.001)

}

}

**Model 7 priors**

In this model, we used vague priors on all parameters as we wanted the prior to be roughly constant in the interval where the likelihood is not zero (18). In this section we use the variance and not precision notation, i.e. $x\sim N\left( mu, s^{2} \right)$ not $x\sim N\left( mu, \Psi\right)$ with the precision $\Psi=s^{-2}$, for all normally distributed priors even though we used precision notation in the OpenBUGS model (18). The following priors were implemented: fixed effects vector $\boldsymbol{\beta}$ for the main and $\boldsymbol{\phi}_{\boldsymbol{i}}$ for $i=\left( 1,\ldots,5 \right)$ imputation sub models had an independent Gaussian distribution i.e. $\boldsymbol{N}\left( 0,1000 \right)$; the scale parameters of the imputation sub models $\boldsymbol{\tau}_{\boldsymbol{\varepsilon}}= \left( \tau_{1\varepsilon}, \tau_{2\varepsilon}, \tau_{3\varepsilon}, \tau_{4\varepsilon} \right)$ denoted as ‘*tau1.int*’, ‘*tau2.int*’‘*taua.int*’ and ‘*taub.int*’, respectively, had conditional conjugate priors with an Inverse-gamma distribution, i.e. $\boldsymbol{IG}\left( 0.001, 0.001 \right)$. The coefficients of the random effects $\boldsymbol{\alpha}=\left( \alpha_{i,1},\alpha_{i,2} \right)$ for each i^th^ patient denoted $U_{i,1}$and $U_{i,2}$ for intercept and slope, respectively, were multivariate normally distributed and had a dispersion parameter denoted ‘*U0*’ for the intercept and slope, which is a product of a normally distributed absolute parameter $\boldsymbol{\Delta}_{\boldsymbol{b}}=\left( \Delta_{1b}, \Delta_{2b} \right)$ denoted as ‘*deltab.abs*’ $\boldsymbol{N}\left( 0, {10}^{6} \right)$, and the respective $\boldsymbol{\vartheta}_{b}=\left( \vartheta_{1b}, \vartheta_{2b} \right)$ denoted ‘*U0.t*’ also absolute and normally distributed $\boldsymbol{N}\left( 0, 1 \right)$. The product makes a distribution of ‘*U0*’ peaked around zero, which forms a favourable distribution for random-effects. The random-effects covariance matrix $\Sigma_{b}$ denoted as ‘*tau*’ follows an Inverse-Wishart distribution, where Wishart distribution i.e. $\boldsymbol{W}\left( R, 3 \right)$ ($R$ is a 2-by-2 diagonal matrix of 0.1) is multiplied by shape parameters $\boldsymbol{\gamma}_{\boldsymbol{b}}\boldsymbol{=}\left( \gamma_{1b}, \gamma_{2b} \right)$ denoted as ‘*D1*’ and ‘*D2*’ for intercept and slope, respectively, and are uniformly distributed $\boldsymbol{U}\left( 0, 100 \right)$.

**Asymptote model with cumulative log viral load – Model 8: cubic B-splines with 3 inner knots**

model{

for( i in 1 : N ) {

for( j in 1 : M ) {

response[i , j] ~ dbern(p[i , j])

p[i , j] <- max(1.0E-5, min(0.99999, ptemp[i , j]))

logit(ptemp[i , j]) <- beta[1] * sex.f[i] + beta[2] * age.count[i] + beta[3] * base.cd4[i] + beta[4] * base.logvl[i] + beta[5] * cumvl[i , j] * cumvlflag[i , j] + b[id[i] , 1] + b[id[i] , 2] * yrs.fu[j] + bspline[1] * time1[i , j] + bspline[2] * time2[i , j] + bspline[3] * time3[i , j] + bspline[4] * time4[i , j] + bspline[5] * time5[i , j] + bspline[6] * time6[i , j] + bspline[7] * time7[i , j] + bspline[8] * time8[i , j]

}

b[i , 1:2] ~ dmnorm(meanb[], tau[ , ])

basecd4.imp[i] <- cut(base.cd4[i])

baselogvl.imp[i] <- cut(base.logvl[i])

base.cd4[i] ~ dnorm(mu1[i], tau1.int)

mu1[i] <- beta1[1] + beta1[2] * sex.f[i] +

beta1[3] * age.count[i]

base.logvl[i] ~ dnorm(mu2[i], tau2.int)

mu2[i] <- beta2[1] + beta2[2] * sex.f[i] +

beta2[3] * age.count[i]

sex.f[i] ~ dbern(phi[i])

phi[i] <- max(1.0E-5, min(0.99999, phitemp[i]))

logit(phitemp[i]) <- beta3[1] +

beta3[2] * age.count[i] + beta3[3] * baselogvl[i] +

beta3[4] * basecd4[i]

}

sigmau0 ~ dunif(0, 100)

sigmau1 ~ dunif(0, 100)

corrb ~ dunif(-1, 1)

sigmaU[1 , 1] <- pow(sigmau0, 2)

sigmaU[2 , 2] <- pow(sigmau1, 2)

sigmaU[1 , 2] <- sigmau0 * sigmau1 * cor

sigmaU[2 , 1] <- sigmaU[1 , 2]

tau[1:2 , 1:2] <- inverse(sigmaU[ , ])

sigma1 <- pow(tau.int,-2)

tau.int ~ dgamma(0.001,0.001)

sigma1.imp <- pow(tau1.int,-2)

tau1.int ~ dgamma(0.001,0.001)

sigma2.imp <- pow(tau2.int,-2)

tau2.int ~ dgamma(0.001,0.001)

sigmaa.imp <- pow(taua.int,-2)

taua.int ~ dgamma(0.001,0.001)

sigmab.imp <- pow(taub.int,-2)

taub.int ~ dgamma(0.001,0.001)

for( k in 1 : 5 ) {

beta[k] ~ dnorm(0, 0.001)

}

for( k in 1 : 3 ) {

beta1[k] ~ dnorm(0, 0.001)

}

for( k in 1 : 3 ) {

beta2[k] ~ dnorm(0, 0.001)

}

for( k in 1 : 4 ) {

beta3[k] ~ dnorm(0, 0.001)

}

for( d in 1 : 8 ) {

bspline[d] ~ dnorm(0, 1.0E-6)

}

}

**Model 8 priors**

In this model, we used vague priors on all parameters as we wanted the prior to be roughly constant in the interval where the likelihood is not zero (18). In this section we use the variance and not precision notation, i.e. $x\sim N\left( mu, s^{2} \right)$ not $x\sim N\left( mu, \Psi\right)$ with the precision $\Psi=s^{-2}$, for all normally distributed priors even though we used precision notation in the OpenBUGS model (18). The following priors were implemented: fixed effects vector $\boldsymbol{\beta}$ for the main and $\boldsymbol{\phi}_{i}$ for $i=\left( 1,\ldots,5 \right)$ imputation sub models had an independent Gaussian distribution i.e. $\boldsymbol{N}\left( 0,1000 \right)$; spline terms *‘bspline’* denoted $\boldsymbol{\theta}$ were also assumed to have an independent Gaussian distribution, i.e. $\boldsymbol{N}\left( 0, {10}^{6} \right)$ because this provides a small precision and would vary less compared to fixed effects; the scale parameters of the imputation sub models $\boldsymbol{\tau}_{\boldsymbol{\varepsilon}}= \left( \tau_{1\varepsilon}, \tau_{2\varepsilon}, \tau_{3\varepsilon}, \tau_{4\varepsilon} \right)$ denoted as ‘*tau1.int*’, ‘*tau2.int*’‘*taua.int*’ and ‘*taub.int*’, respectively, had conditional conjugate priors with an Inverse-gamma distribution, i.e. $\boldsymbol{IG}\left( 0.001, 0.001 \right)$; for random-effects covariance matrix $\Sigma_{b}$ denoted as ‘*tau*’, we specified uniform distributions for the standard deviations $\sigma_{0}$ denoted ‘*sigmau0*’ and $\sigma_{1}$ denoted ‘*sigmau1*’for random intercept and slope i.e. $\boldsymbol{U}\left( 0, 100 \right)$; and for the correlation $\rho$ denoted as *‘cor’* a Uniform distribution $\boldsymbol{U}\left( 1, -1 \right)$.

**Asymptote model with cumulative log viral load – Model 9: cubic B-splines with 5 inner knots**

model{

for( i in 1 : N ) {

for( j in 1 : M ) {

response[i , j] ~ dbern(p[i , j])

p[i , j] <- max(1.0E-5, min(0.99999, ptemp[i , j]))

logit(ptemp[i , j]) <- beta[1] * sex.f[i] + beta[2] * age.count[i] + beta[3] * base.cd4[i] + beta[4] * base.logvl[i] + beta[5] * cumvl[i , j] * cumvlflag[i , j] + b[id[i] , 1] + b[id[i] , 2] * yrs.fu[j] + bspline[1] * time1[i , j] + bspline[2] * time2[i , j] + bspline[3] * time3[i , j] + bspline[4] * time4[i , j] + bspline[5] * time5[i , j] + bspline[6] * time6[i , j] + bspline[7] * time7[i , j] + bspline[8] * time8[i , j] + bspline[9] * time9[i , j] + bspline[10] * time10[i , j]

}

b[i , 1:2] ~ dmnorm(meanb[], tau[ , ])

basecd4.imp[i] <- cut(base.cd4[i])

baselogvl.imp[i] <- cut(base.logvl[i])

base.cd4[i] ~ dnorm(mu1[i], tau1.int)

mu1[i] <- beta1[1] + beta1[2] * sex.f[i] +

beta1[3] * age.count[i]

base.logvl[i] ~ dnorm(mu2[i], tau2.int)

mu2[i] <- beta2[1] + beta2[2] * sex.f[i] +

beta2[3] * age.count[i]

sex.f[i] ~ dbern(phi[i])

phi[i] <- max(1.0E-5, min(0.99999, phitemp[i]))

logit(phitemp[i]) <- beta3[1] +

beta3[2] * age.count[i] + beta3[3] * baselogvl[i] +

beta3[4] * basecd4[i]

}

sigmau0 ~ dunif(0, 100)

sigmau1 ~ dunif(0, 100)

corrb ~ dunif(-1, 1)

sigmaU[1 , 1] <- pow(sigmau0, 2)

sigmaU[2 , 2] <- pow(sigmau1, 2)

sigmaU[1 , 2] <- sigmau0 * sigmau1 * cor

sigmaU[2 , 1] <- sigmaU[1 , 2]

tau[1:2 , 1:2] <- inverse(sigmaU[ , ])

sigma1 <- pow(tau.int,-2)

tau.int ~ dgamma(0.001,0.001)

sigma1.imp <- pow(tau1.int,-2)

tau1.int ~ dgamma(0.001,0.001)

sigma2.imp <- pow(tau2.int,-2)

tau2.int ~ dgamma(0.001,0.001)

sigmaa.imp <- pow(taua.int,-2)

taua.int ~ dgamma(0.001,0.001)

sigmab.imp <- pow(taub.int,-2)

taub.int ~ dgamma(0.001,0.001)

for( k in 1 : 5 ) {

beta[k] ~ dnorm(0, 0.001)

}

for( k in 1 : 3 ) {

beta1[k] ~ dnorm(0, 0.001)

}

for( k in 1 : 3 ) {

beta2[k] ~ dnorm(0, 0.001)

}

for( k in 1 : 4 ) {

beta3[k] ~ dnorm(0, 0.001)

}

for( d in 1 : 10 ) {

bspline[d] ~ dnorm(0, 1.0E-6)

}

}

**Model 9 priors**

In this model, we used vague priors on all parameters as we wanted the prior to be roughly constant in the interval where the likelihood is not zero (18). In this section we use the variance and not precision notation, i.e. $x\sim N\left( mu, s^{2} \right)$ not $x\sim N\left( mu, \Psi\right)$ with the precision $\Psi=s^{-2}$, for all normally distributed priors even though we used precision notation in the OpenBUGS model (18). The following priors were implemented: fixed effects vector $\boldsymbol{\beta}$ for the main and $\boldsymbol{\phi}_{\boldsymbol{i}}$ for $i=\left( 1,\ldots,5 \right)$ imputation sub models had an independent Gaussian distribution i.e. $\boldsymbol{N}\left( 0,1000 \right)$; spline terms *‘bspline’* denoted $\boldsymbol{\theta}$ were also assumed to have an independent Gaussian distribution, i.e. $\boldsymbol{N}\left( 0, {10}^{6} \right)$ because this provides a small precision and would vary less compared to fixed effects; the scale parameters of the imputation sub models $\boldsymbol{\tau}_{\boldsymbol{\varepsilon}}= \left( \tau_{1\varepsilon}, \tau_{2\varepsilon}, \tau_{3\varepsilon}, \tau_{4\varepsilon} \right)$ denoted as ‘*tau1.int*’, ‘*tau2.int*’‘*taua.int*’ and ‘*taub.int*’, respectively, had conditional conjugate priors with an Inverse-gamma distribution, i.e. $\boldsymbol{IG}\left( 0.001, 0.001 \right)$; for random-effects covariance matrix $\Sigma_{b}$ denoted as ‘*tau*’, we specified uniform distributions for the standard deviations $\sigma_{0}$ denoted ‘*sigmau0*’ and $\sigma_{1}$ denoted ‘*sigmau1*’ for random intercept and slope i.e. $\boldsymbol{U}\left( 0, 100 \right)$; and for the correlation $\rho$ denoted as *‘cor’* a Uniform distribution $\boldsymbol{U}\left( 1, -1 \right)$.
