# Supplementary material for: Cumulative viral load as a predictor of CD4+ T-cell response to antiretroviral therapy using Bayesian statistical models
Source: PLoS One. 2019 Nov 13;14(11):e0224723. doi: 10.1371/journal.pone.0224723 (PMC6853324; doi:10.1371/journal.pone.0224723)
Supplement: S2 Appendix — (DOCX) [file pone.0224723.s002.docx]

**S2 Appendix**

**Figure A: Data selection diagram**

**
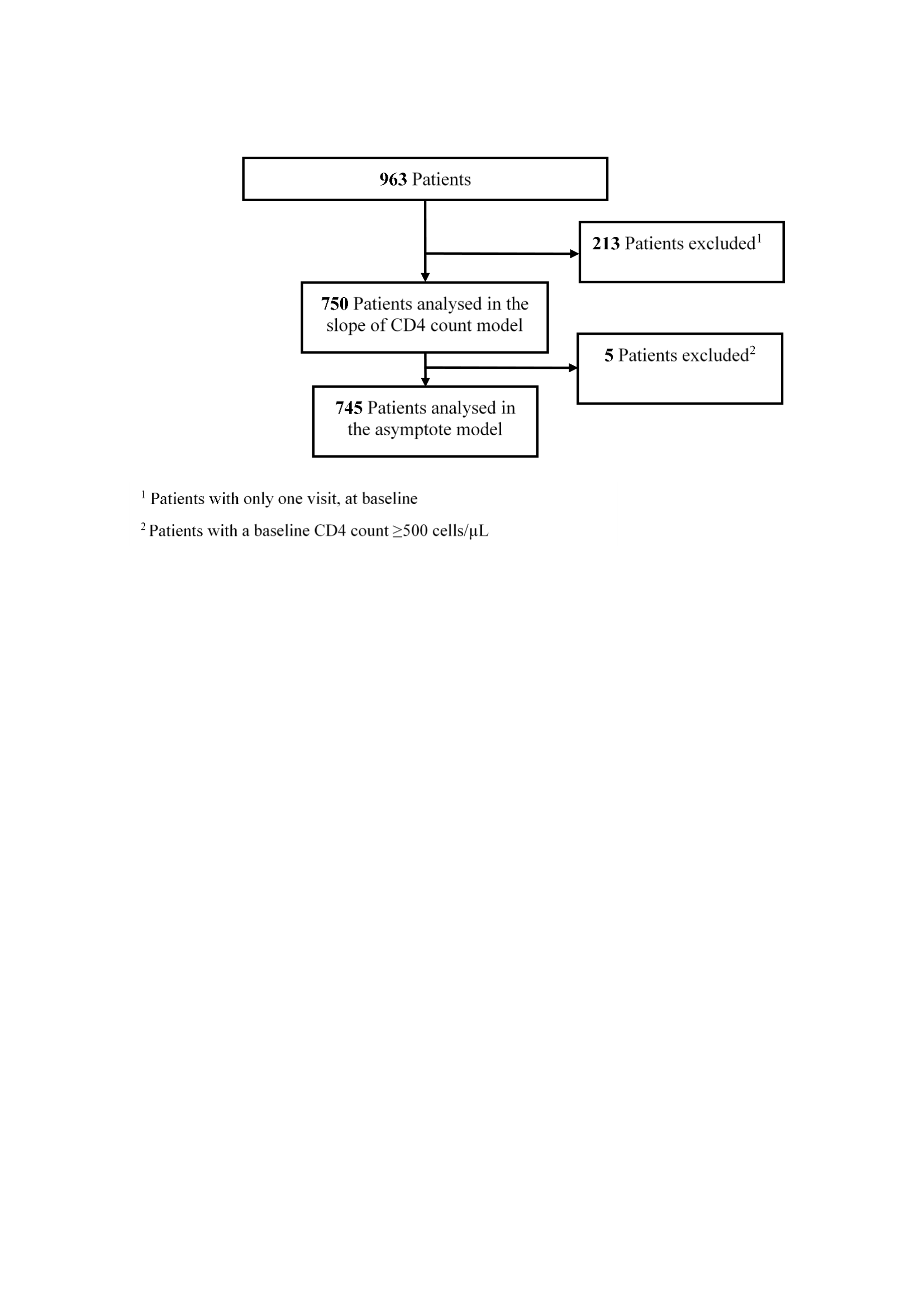
**

**Figure B: Proportion of missing viral load result per visit over throughout follow-up period.**

**
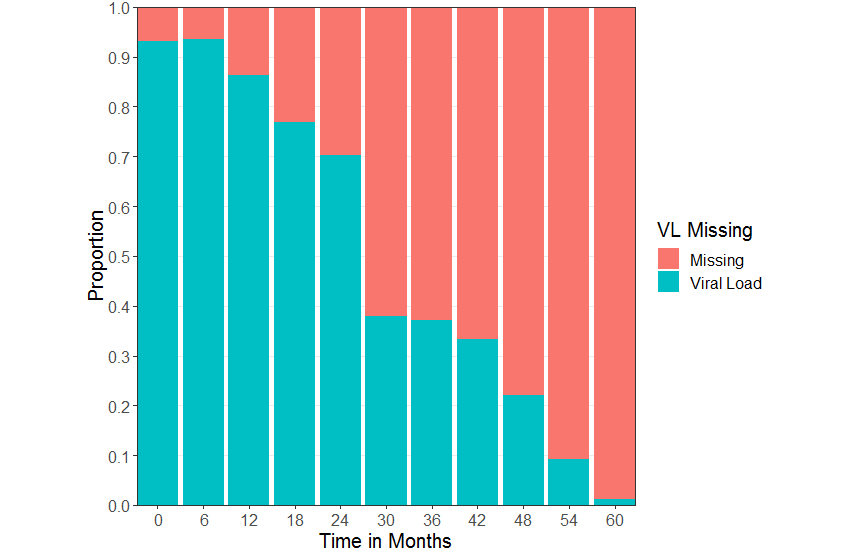
**

Figure C: Histograms of estimated random intercept and slope obtained for the slope of CD4 count polynomial models


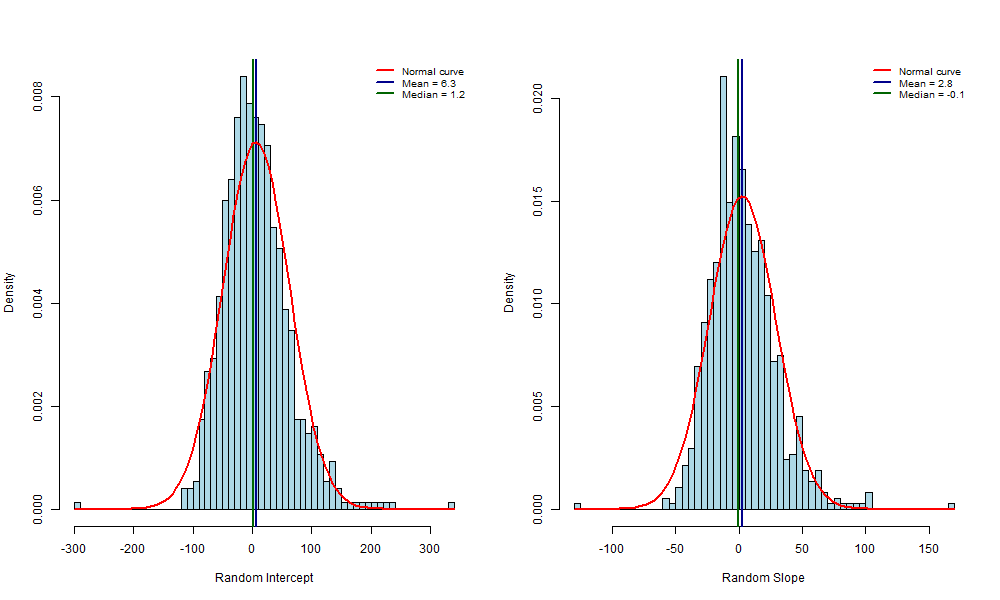


Legend: the red-line is the distribution super imposed on the random intercet and slope histograms. The blue-line and green-line is for mean and median of the distributions.

Figure 4: Histograms of estimated random intercept and slope obtained from the asymptote model


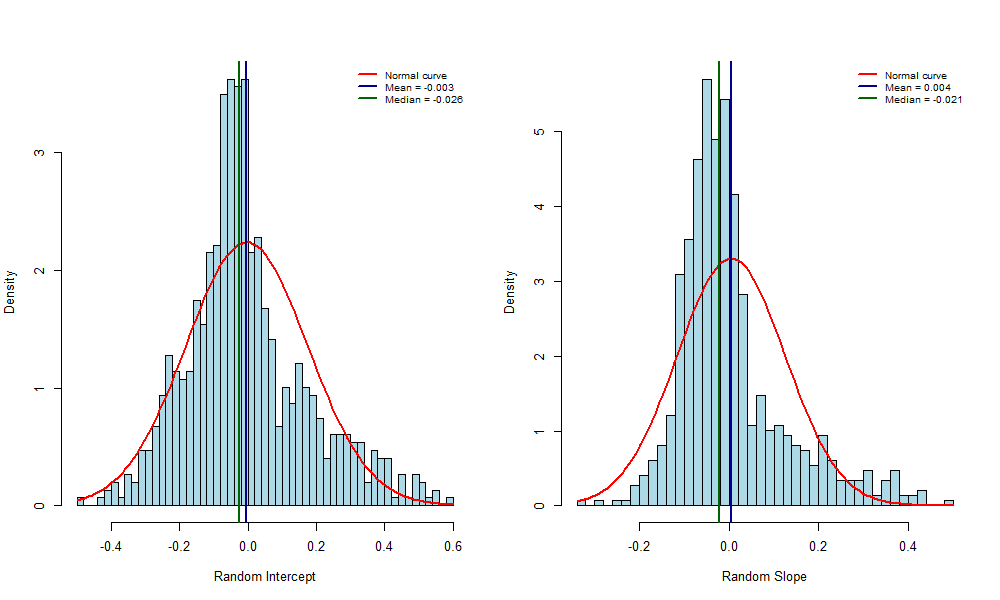


Legend: **the red-line is the distribution super imposed on the random intercet and slope histograms. The blue-line and green-line is for mean and median of the distributions**

**Figure D: Cross correlation plots between corresponding parameters adjusted for in the primary models**


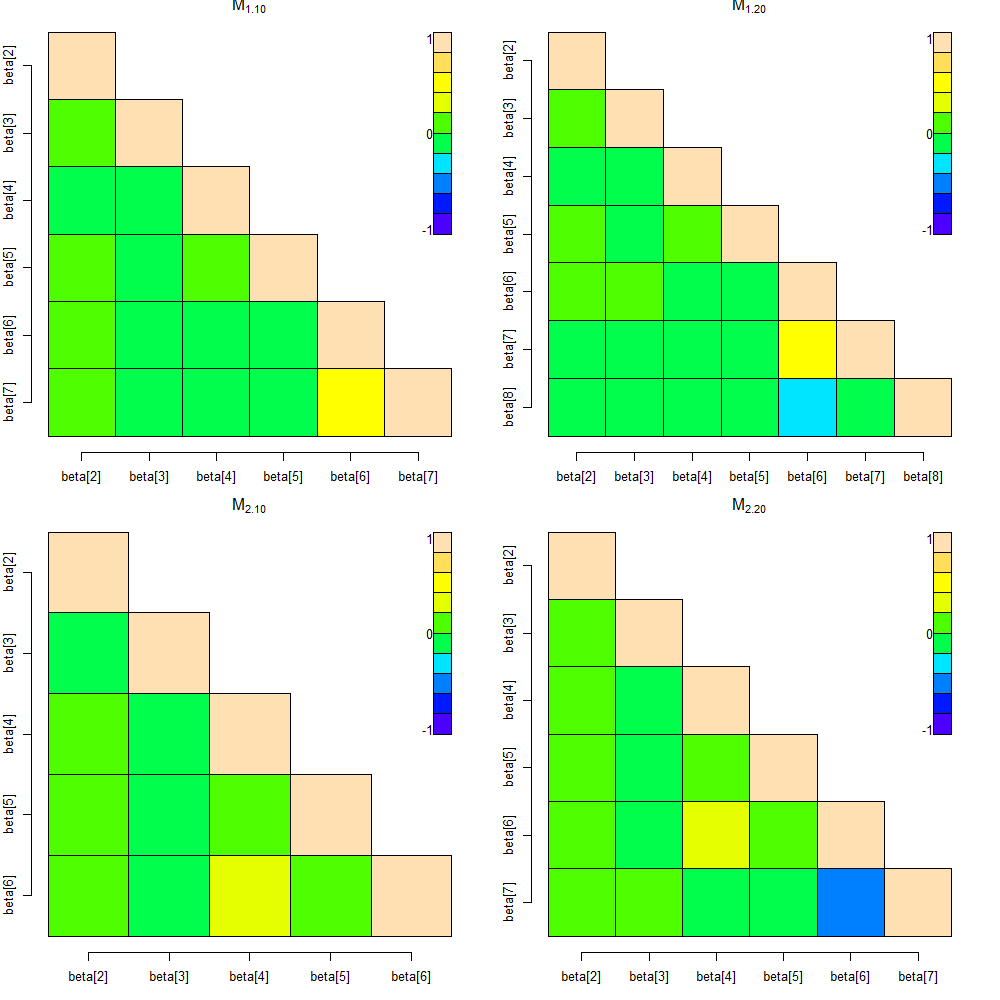


**Legend: *M_1.10_* and *M_1.20_*** (referred to as Model 1 in the text) are models without or with cumulative log viral load, respectively. In these models, CD4 counts outcome in the likelihood follows normal distribution, while beta priors follow independent Gaussian normal distribution; and ***M_2.10_*** and ***M_2.20_*** ((referred to as Model 6 in the text) are models without or with cumulative log viral load, respectively. In these models, are having ≥500 cells/µL follows Bernoulli distribution, beta priors follow independent Gaussian normal distribution. Beta[2] up to beta[6] represents sex, baseline age, baseline CD4 count, baseline log viral load and time on treatment. For ***M_1.10_*** and ***M_1.20_*** ((referred to as Model 1 in the text) beta[7] and beta[8] are time on treatment-squared and cumulative log viral load, respectively, while beta[7] was for cumulative log viral load in ***M_2.10_*** and ***M_2.20_*** (referred to as Model 6 in the text) and beta[6] is cumulative log viral load.

| **Parameter** | **Model without cVL_2_** | | **Model with cVL_2_** | |
| --- | --- | --- | --- | --- |
|  | Estimate | 95% CI | Estimate | 95% CI |
| **Female-sex** | 17.3 | (6.00, 28.55) | 17.3 | (6.16, 28.42) |
| **Baseline age** | -6.9 | (-12.14, -1.69) | -6.9 | (-12.03, -1.70) |
| **Baseline CD4 count** | 83.8 | (78.30, 89.32) | 83.8 | (78.31, 89.29) |
| **Baseline log viral load** | 6.7 | (1.37, 12.11) | 6.8 | (1.42, 12.06) |
| **cumulative log viral load** | – | – | -0.4 | (-9.46, 8.65) |

**Table A: Implementing the model with cubic splines and 3 inner knots for the slope of CD4 counts model with posterior mean and 95% credible intervals.**

**Legend:** **cVL_2_**—cumulative log viral load

**Table B: Effect of using cubic splines with 5 inner knots for the slope of CD4 counts model with posterior mean and 95% credible intervals.**

| **Parameter** | **Model without cVL_2_** | | **Model with cVL_2_** | |
| --- | --- | --- | --- | --- |
|  | Estimate | 95% CI | Estimate | 95% CI |
| **Female-sex** | 17.42 | (6.27, 28.61) | 17.0 | (5.71, 28.25) |
| **Baseline age** | -6.96 | (-12.10, -1.82) | -6.7 | (-11.99, -1.47) |
| **Baseline CD4 count** | 83.72 | (78.22, 89.19) | 84.0 | (78.42, 89.57) |
| **Baseline log viral load** | 6.83 | (1.44, 12.25) | 6.82 | (1.38, 12.31) |
| **cumulative log viral load** | – | – | -0.82 | (-9.81, 8.27) |

**Legend:** **cVL_2_**—cumulative log viral load

| **Parameter** | **Model without cVL_2_** | | **Model with cVL_2_** | |
| --- | --- | --- | --- | --- |
|  | Estimate | 95% CI | Estimate | 95% CI |
| **Female-sex** | 24.2 | (13.12, 35.35) | 23.8 | (12.61, 35.12) |
| **Baseline age** | -6.9 | (-12.10, -1.70) | -6.4 | (-11.61, -1.14) |
| **Baseline CD4 count** | 82.3 | (76.89, 87.73) | 82.8 | (77.36, 88.29) |
| **Baseline log viral load** | 7.5 | (2.17, 12.91) | 7.5 | (2.06, 12.91) |
| **Time on treatment** | 52.3 | (45.51, 59.06) | 55.7 | (48.72, 62.57) |
| **Time on treatment-squared** | -23.0 | (-25.00, -20.91) | -22.8 | (-24.84, -20.78) |
| **cumulative log viral load** | – | – | **-19.4** | **(-28.12, -10.74)** |

**Table C: Effect of using skew-normal random effect in the slope of CD4 count model with posterior mean and 95% credible intervals**

**Legend:** **cVL_2_**—cumulative log viral load

**Table D: Effect of using informative priors in the slope of CD4 count model where parameters followed Gaussian normal distribution with posterior mean and 95% credible intervals.**

| **Parameter** | **Model *1*** | | **Model *1^*^*** | |
| --- | --- | --- | --- | --- |
|  | Estimate | 95% CI | Estimate | 95% CI |
| **Female-sex** | 23.8 | (12.53, 34.96) | 24.6 | (13.31, 35.92) |
| **Baseline age** | -6.3 | (-11.57, -1.08) | -6.3 | (-11.55, -1.06) |
| **Baseline CD4 count** | 82.9 | (77.4, 88.38) | 82.8 | (77.39, 88.28) |
| **Baseline log viral load** | 7.5 | (2.03, 12.92) | 7.6 | (2.12, 13.05) |
| **Time on treatment** | 55.7 | (48.83, 62.65) | 56.5 | (49.54, 63.30) |
| **Time on treatment-squared** | -22.7 | (-24.86, -20.78) | -22.7 | (-24.75, -20.68) |
| **cumulative log viral load** | **-19.6** | **(-28.25, -10.93)** | **-19.7** | **(-28.35, -11.10)** |

**Legend:** ***model 1*** – Gaussian normally distributed CD4 count in the likelihood; *and* ***model 1*^*^** – model 1 but with informative priors.

**Table E: Effect of using skew-normal for random effect in the asymptote model with posterior odds ratios and 95% credible intervals.**

| **Parameter** | **Model without cVL_2_** | | **Model with cVL_2_** | |
| --- | --- | --- | --- | --- |
|  | Estimate | 95%CI | Estimate | 95%CI |
| **Female-sex** | 6.26 | (2.992, 13.902) | 6.52 | (3.004, 14.397) |
| **Baseline age** | 0.54 | (0.393, 0.744) | 0.54 | (0.386, 0.748) |
| **Baseline CD4 count** | 2.90 | (2.141, 3.995) | 3.04 | (2.235, 4.242) |
| **Baseline log viral load** | 1.25 | (0.900, 1.742) | 1.27 | (0.904, 1. 785) |
| **Time on treatment** | 3.49 | (2.670, 4.536) | 4.04 | (3.034, 5.425) |
| **Time on treatment - squared** | 0.67 | (0.582, 0.759) | 0.65 | (0.564, 0.746) |
| **cumulative log viral load** | – | – | **0.41** | **(0.236, 0.718)** |

**Legend:** **cVL_2_**—cumulative log viral load

**Table F: Effect of using cubic splines with 3 inner knots in the asymptote model with posterior odds ratios and 95% credible intervals.**

| **Parameter** | **Model without cVL_2_** | | **Model with cVL_2_** | |
| --- | --- | --- | --- | --- |
|  | Estimate | 95%CI | Estimate | 95%CI |
| **Female-sex** | 4.62 | (2.360, 9.450) | 4.62 | (2.377, 9. 545) |
| **Baseline age** | 0.64 | (0.486, 0.826) | 0.64 | (0.484, 0.834) |
| **Baseline CD4 count** | 2.32 | (1.864, 2.912) | 2.38 | (1.892, 2.995) |
| **Baseline log viral load** | 1.12 | (0.846, 1.454) | 1.11 | (0.842, 1.459) |
| **cumulative log viral load** | **–** | **–** | **0.76** | **(0.465, 1.244)** |

**Legend:** **cVL_2_**—cumulative log viral load

**Table G: Effect of using cubic splines with 5 inner knots in the asymptote model with posterior odds ratios and 95% credible intervals.**

| **Parameter** | **Model without cVL_2_** | | **Model with cVL_2_** | |
| --- | --- | --- | --- | --- |
|  | Estimate | 95%CI | Estimate | 95%CI |
| **Female-sex** | 4.67 | (2.358, 9.875) | 4.74 | (2.386, 9.526) |
| **Baseline age** | 0.63 | (0.483, 0.828) | 0.64 | (0.486, 0.874) |
| **Baseline CD4 count** | 2.33 | (1.855, 2.936) | 2.35 | (1.856, 2.977) |
| **Baseline log viral load** | 1.11 | (0.849, 1.459) | 1.11 | (0.841, 1.472) |
| **cumulative log viral load** | **–** | **–** | **0.77** | **(0.473, 1.276)** |

**Legend:** **cVL_2_**—cumulative log VL

Table H: Model selection for slope of CD4 count and asymptote models

| Model - | without cVL_2_ | with cVL_2_ |
| --- | --- | --- |
|  | cDIC | cDIC |
| Slope models |  |  |
| Model 1 | 47510 | 47480 |
| Model 2 | 48120 | 48130 |
| Model 3 | 48120 | 48130 |
| Model 4 | 47270 | 47330 |
| Model 5 | -3186 | -29310 |
| Asymptote models |  |  |
| Model 6 | 1716 | 1695 |
| Model 7 | 1108 | 829 |
| Model 8 | 1701 | 1695 |
| Model 9 | 1702 | 1689 |

**Legend: *model 1***—models errors and random effects are normally distributed; ***model 2***—models errors and random effects are normally distributed and has cubic splines with 3 inner knots; ***model 3***—models errors and random effects are normally distributed and has cubic splines with 5 inner knots; ***model 4***—random-effects are ***SN*** distributed; ***model 5***— measurement error and random-effects are ***SN*** distributed; ***model 6***—random effects are normally distributed; ***model 7*** –random effects are skew-normally distributed; ***model 8***—random-effects are normally distributed with cubic splines and 3 inner knots; ***model 9***—random-effects are normally distributed with cubic splines and 5 inner knots.
